# Supplementary material for: The Causal Relationship between Inflammatory Cytokines and Liver Cirrhosis in European Descent: A Bidirectional Two-Sample Mendelian Randomization Study and the First Conclusions
Source: Biomedicines. 2024 Oct 4;12(10):2264. doi: 10.3390/biomedicines12102264 (PMC12365905; doi:10.3390/biomedicines12102264)
Supplement: Supplementary file 1 [file biomedicines-12-02264-s001.zip › Supplementary Figures.pdf]

Supplementary Figures

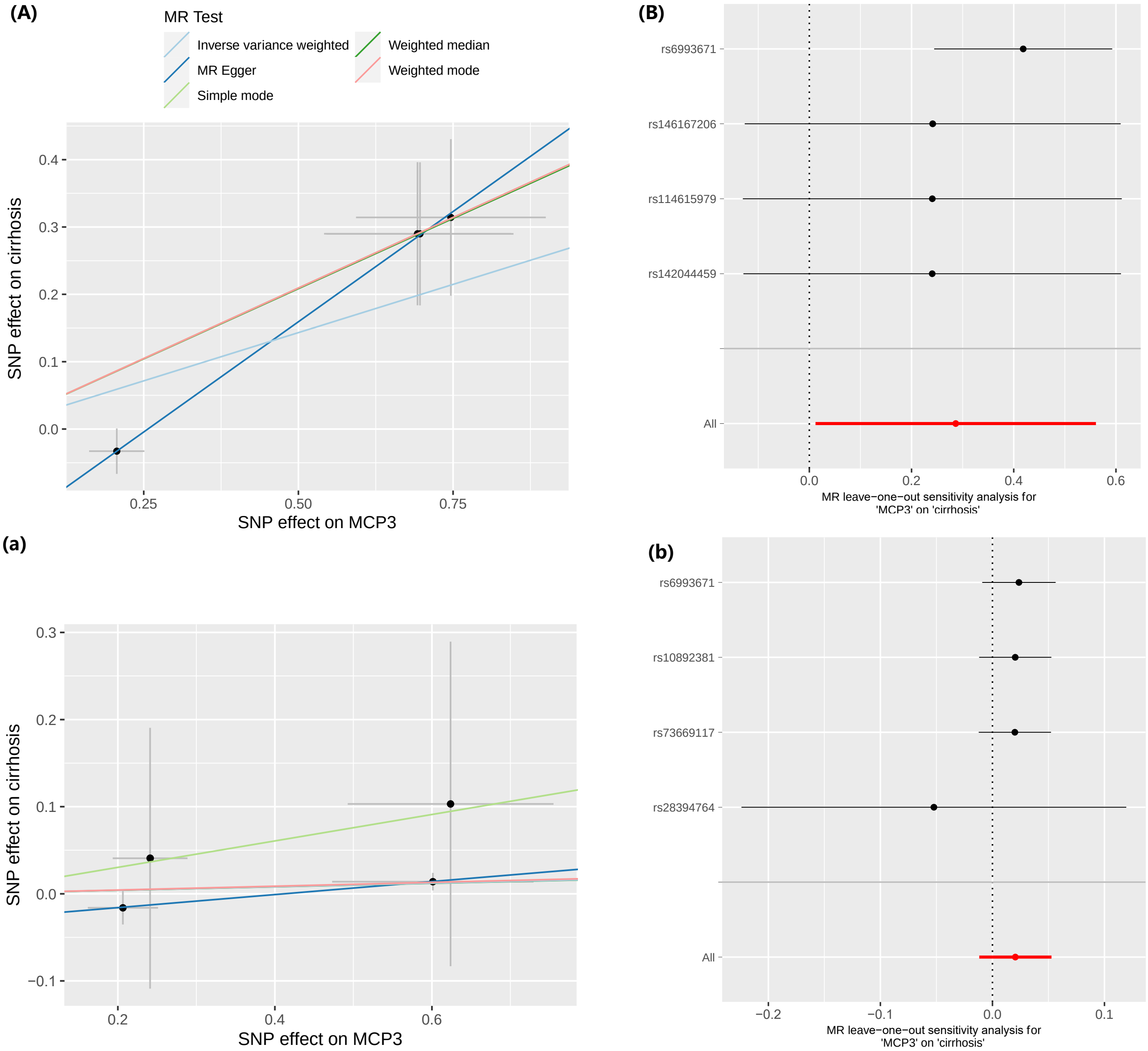

Figure S1. MR Plots for Relationship of MCP-3 with cirrhosis. MCP-3, Monocyte specific chemokine 3; MR, mendelian randomization; (A) Scatter plot of single-nucleotide polymorphism (SNP) effects on MCP-3 vs cirrhosis for Dataset 1, with the slope of each line corresponding to estimated MR effect per method. (B) Forest plot of individual and combined SNP MR-estimated effect sizes for Dataset 1. (a) Scatter plot of single-nucleotide polymorphism (SNP) effects on MCP-3 vs cirrhosis for Dataset 2, with the slope of each line corresponding to estimated MR effect per method. (b) Forest plot of individual and combined SNP MR-estimated effect sizes for Dataset 2.

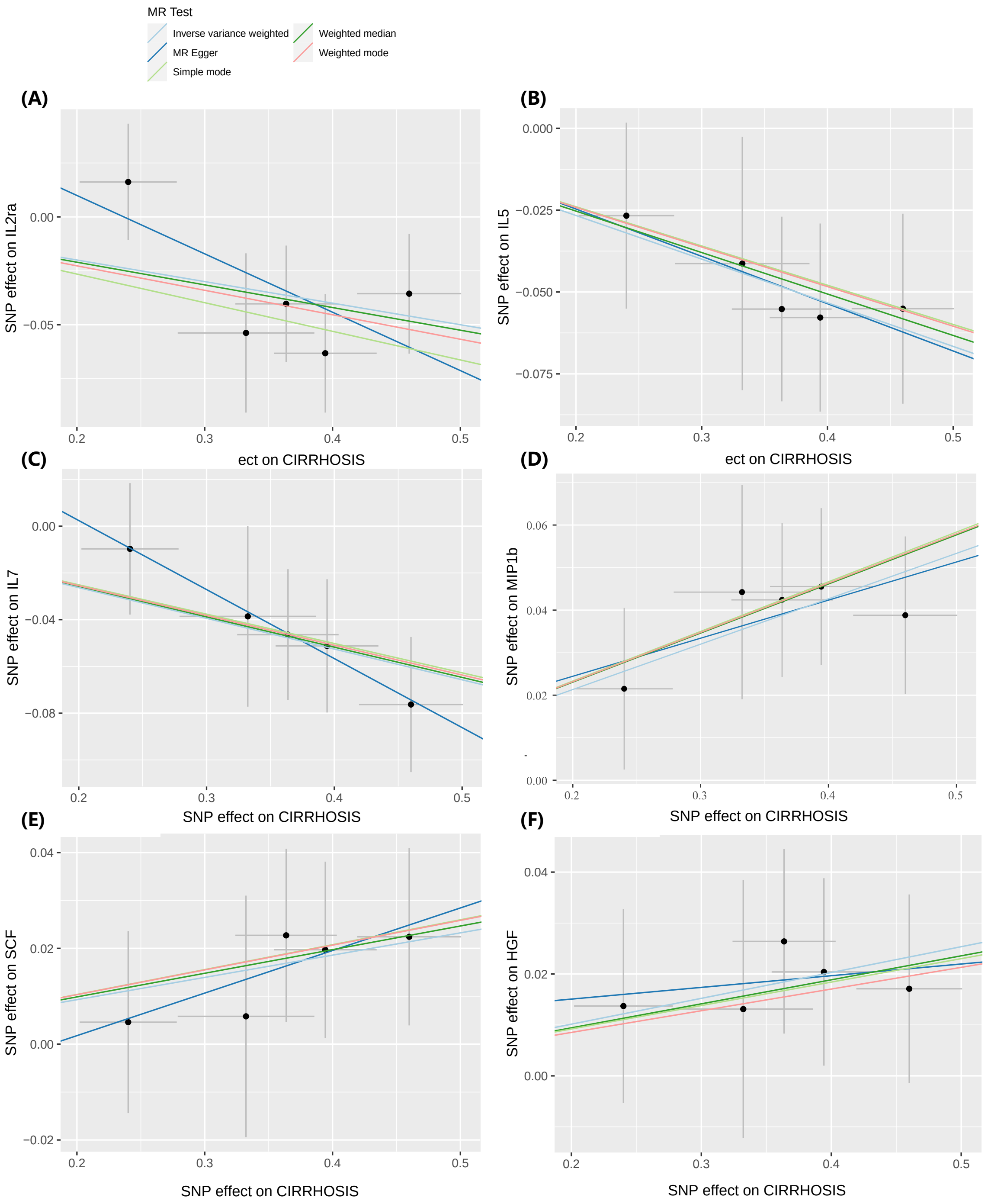

Figure S2. Scatter plot of single-nucleotide polymorphism (SNP) effects on cirrhosis vs IL-2ra, IL5, IL7, MIP1b, SCF, and HGF for Dataset 1, with the slope of each line corresponding to estimated MR effect per method. IL-2ra，Interleukin-2 receptor antagonist；IL5, Interleukin-5; IL7, Interleukin-7; MIP1b, Macrophage inflammatory protein 1b; MR, mendelian randomization; SCF, Stem cell factor; HGF, Hepatocyte growth factor.

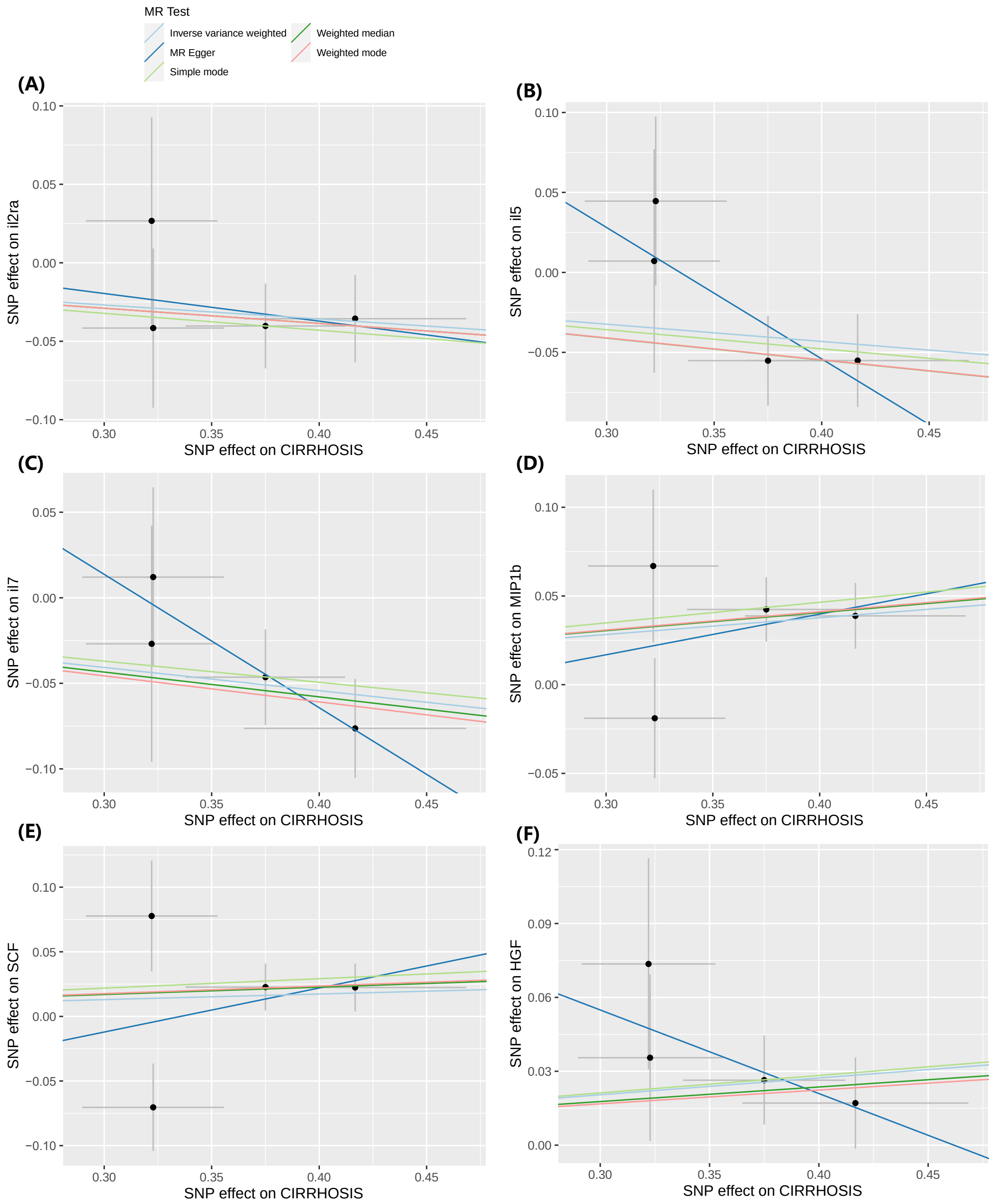

Figure S3. Scatter plot of single-nucleotide polymorphism (SNP) effects on cirrhosis vs IL-2ra, IL5, IL7, MIP1b, SCF, and HGF for Dataset 2, with the slope of each line corresponding to estimated MR effect per method. IL-2ra，Interleukin-2 receptor antagonist；IL5, Interleukin-5; IL7, Interleukin-7; MIP1b, Macrophage inflammatory protein 1b; MR, mendelian randomization; SCF, Stem cell factor; HGF, Hepatocyte growth factor.

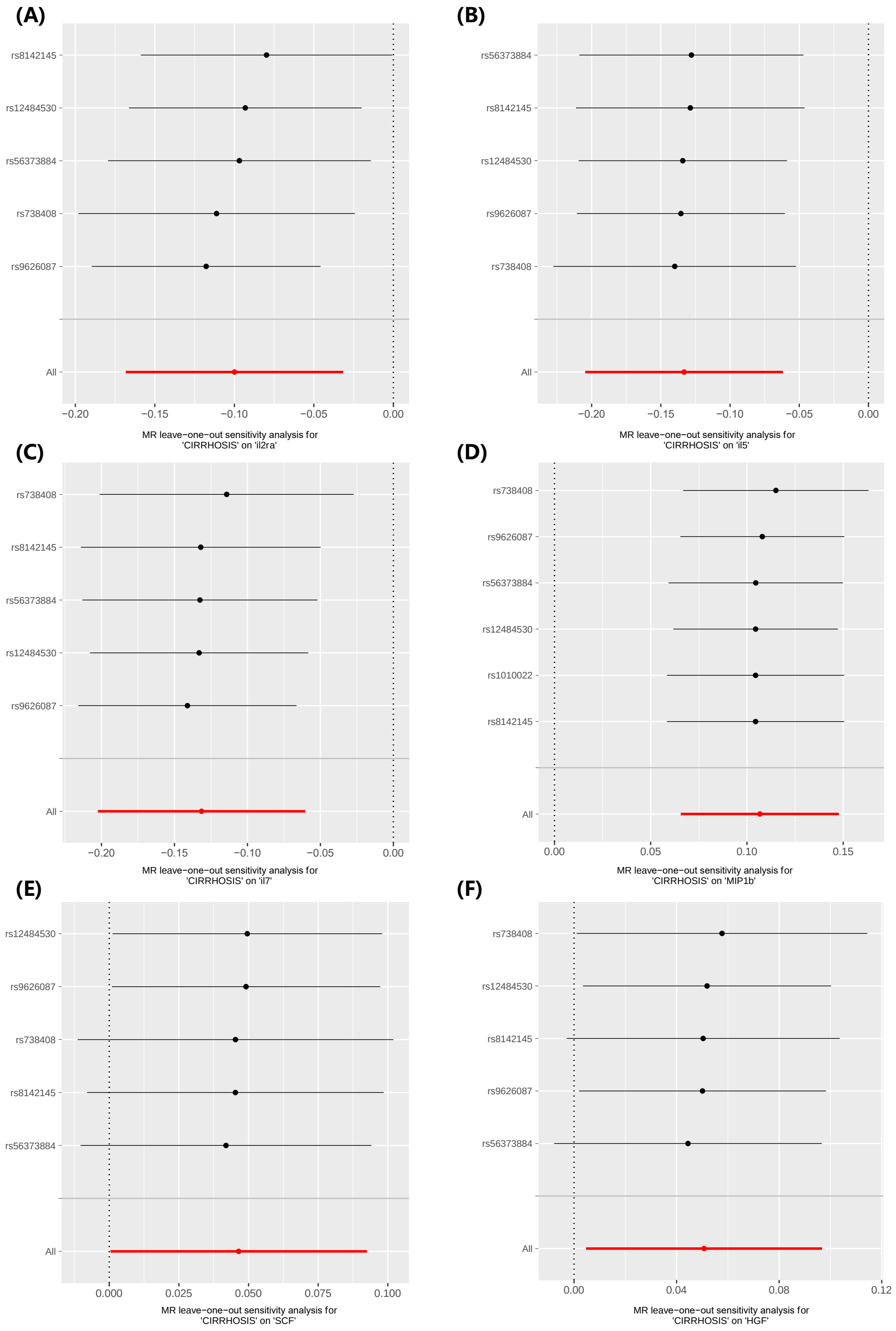

Figure S4. Plots of leave-one-out analyses to assess the stability of inverse cirrhosis results for Dataset 1.

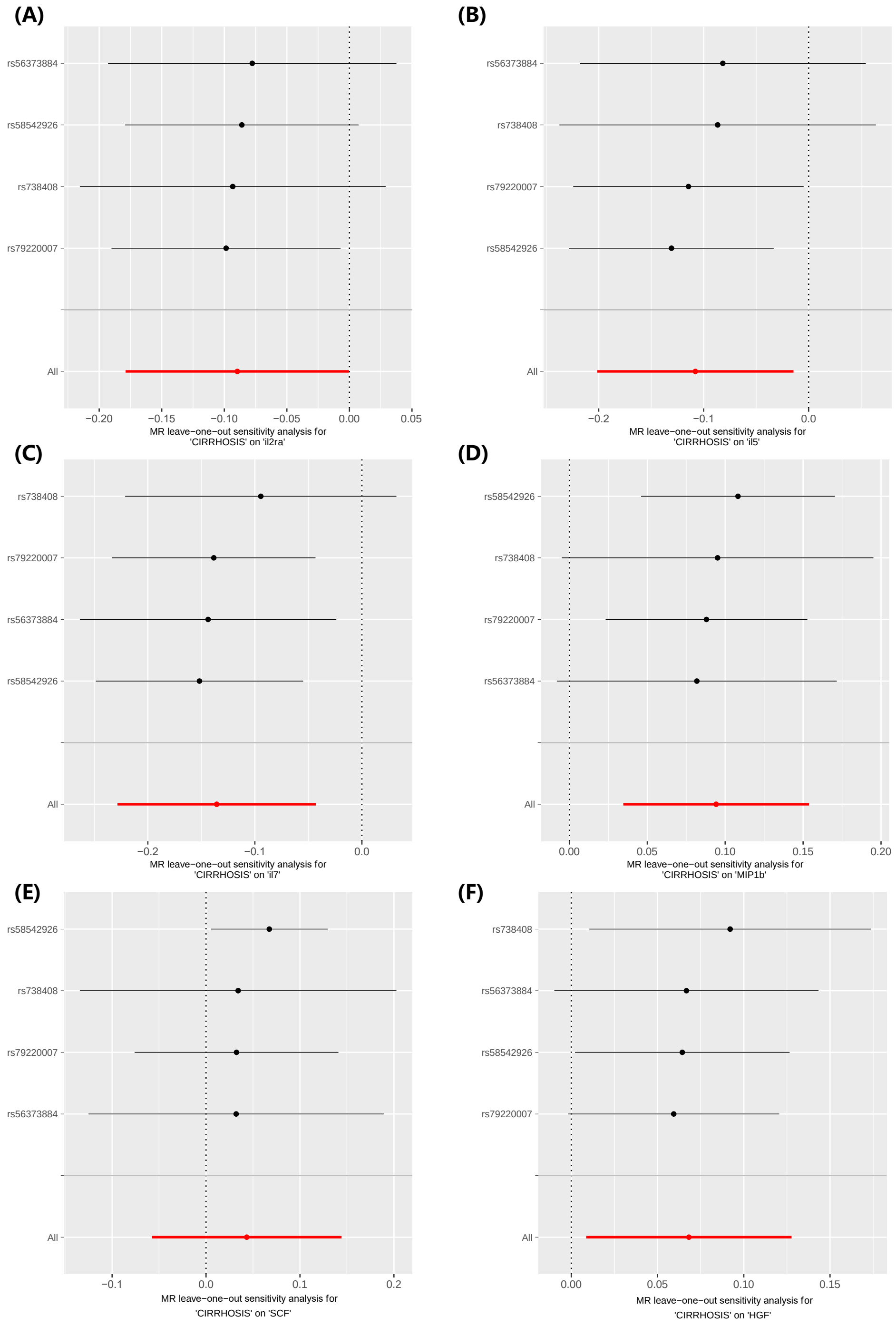

Figure S5. Plots of leave-one-out analyses to assess the stability of inverse cirrhosis results for Dataset 2.
