# Supplementary material for: The Causal Relationship between Inflammatory Cytokines and Liver Cirrhosis in European Descent: A Bidirectional Two-Sample Mendelian Randomization Study and the First Conclusions
Source: Biomedicines. 2024 Oct 4;12(10):2264. doi: 10.3390/biomedicines12102264 (PMC12365905; doi:10.3390/biomedicines12102264)
Supplement: Supplementary file 1 [file biomedicines-12-02264-s001.zip › Supplementary Tables.pdf]

Supplementary Table

Supplementary Table S1. MR estimates for Dataset 1.

Category	Exposure	No. of SNPs	IVW			MR-Lasso			MR-Egger			Weighted Median			Simple Mode			Weighted Mode		
			OR	95% CI	pval	OR	95% CI	pval	OR	95% CI	pval	OR	95% CI	pval	OR	95% CI	pval	OR	95% CI	pval
**Chemokines**																				
	CTACK	7	0.810	(0.617,1.065)	0.131	0.877	(0.694,1.110)	0.275	1.148	(0.600,2.195)	0.694	0.998	(0.721,1.382)	0.992	1.064	(0.634,1.787)	0.822	1.044	(0.660,1.651)	0.860
	Eotaxin	11	0.856	(0.680-1.077)	0.183	0.957	(0.876,1.045)	0.324	0.567	(0.350,0.918)	0.046	0.746	(0.554,1.005)	0.054	0.752	(0.474,1.193)	0.254	0.721	(0.507,1.025)	0.099
	GROa	6	0.971	(0.785,1.201)	0.785	0.946	(0.783,1.145)	0.570	0.850	(0.496,1.457)	0.756	1.044	(0.798,1.365)	0.756	1.096	(0.720,1.669)	0.685	1.091	(0.775,1.536)	0.639
	IP10	3	0.707	(0.516,0.969)	0.031	0.939	(0.661,1.335)	0.726	0.835	(0.476,1.464)	0.642	0.727	(0.494,1.072)	0.108	0.728	(0.461,1.152)	0.308	0.762	(0.475,1.223)	0.377
	MCP1	10	0.794	(0.600,1.051)	0.107	0.824	(0.637,1.068)	0.144	0.546	(0.322,0.926)	0.055	0.691	(0.510,0.938)	0.018	0.645	(0.409,1.016)	0.091	0.664	(0.430,1.027)	0.099
	MCP3	4	1.332	(1.012,1.752)	0.041	1.516	(1.298,1.768)	<0.000	1.925	(1.457,2.542)	0.044	1.518	(1.225,1.881)	0.000	1.520	(1.187,1.948)	0.045	1.520	(1.167,1.981)	0.053
	MIG	13	1.123	(0.966,1.305)	0.131	1.057	(0.928,1.203)	0.408	1.240	(0.915,1.681)	0.193	1.066	(0.873,1.303)	0.528	1.134	(0.835,1.540)	0.437	1.143	(0.829,1.576)	0.432
	MIP1α	5	0.812	(0.631,1.044)	0.104	0.861	(0.688,1.078)	0.191	1.143	(0.529,2.470)	0.756	0.797	(0.578,1.097)	0.164	0.769	(0.483,1.223)	0.329	0.771	(0.484,1.227)	0.334
	MIP1β	17	0.989	(0.821,1.191)	0.910	0.972	(0.802,1.179)	0.779	1.433	(0.900,2.281)	0.151	0.968	(0.744,1.258)	0.808	0.949	(0.604,1.491)	0.823	0.949	(0.588,1.530)	0.832
	RANTES	9	0.889	(0.740,1.068)	0.209	0.963	(0.801,1.156)	0.682	0.761	(0.468,1.238)	0.308	0.952	(0.750,1.209)	0.687	0.973	(0.659,1.435)	0.892	0.994	(0.678,1.457)	0.976
	SDF1α	7	1.039	(0.742,1.454)	0.825	0.981	(0.716,1.344)	0.905	0.866	(0.495,1.515)	0.635	0.929	(0.606,1.424)	0.736	0.915	(0.532,1.573)	0.758	0.898	(0.528,1.528)	0.705
**Growthfactors**																				
	bNGF	6	1.100	(0.885,1.367)	0.389	1.063	(0.868,1.301)	0.553	1.966	(0.651,5.938)	0.297	1.131	(0.858,1.491)	0.382	1.106	(0.716,1.710)	0.669	1.124	(0.733,1.725)	0.615
	FGFBasic	6	0.797	(0.571,1.112)	0.182	0.891	(0.665,1.194)	0.439	0.843	(0.370,1.921)	0.705	0.877	(0.581,1.322)	0.530	0.912	(0.507,1.640)	0.771	0.909	(0.511,1.615)	0.758
	GCSF	9	1.039	(0.805,1.341)	0.769	1.123	(0.896,1.406)	0.315	1.230	(0.808,1.872)	0.367	1.022	(0.720,1.453)	0.902	1.069	(0.611,1.870)	0.820	0.990	(0.591,1.658)	0.970
	HGF	8	1.179	(0.897,1.550)	0.238	1.166	(0.913,1.492)	0.218	1.556	(0.823,2.944)	0.223	1.135	(0.802,1.606)	0.475	1.157	(0.686,1.953)	0.601	1.124	(0.706,1.789)	0.638
	MCSF	8	0.990	(0.863,1.136)	0.889	1.015	(0.889,1.161)	0.822	0.875	(0.686,1.115)	0.321	0.983	(0.819,1.180)	0.857	1.043	(0.760,1.431)	0.802	1.006	(0.768,1.317)	0.968
	PDGFbb	11	1.267	(0.946,1.698)	0.113	1.184	(0.909,1.542)	0.209	1.420	(0.683,2.953)	0.373	1.222	(0.856,1.744)	0.270	0.892	(0.454,1.756)	0.748	0.915	(0.485,1.726)	0.789
	SCF	10	0.968	(0.744,1.259)	0.808	1.031	(0.803,1.326)	0.806	0.862	(0.463,1.605)	0.652	0.949	(0.682,1.319)	0.755	0.959	(0.597,1.540)	0.867	0.957	(0.580,1.580)	0.867
	SCGFβ	15	1.005	(0.882,1.145)	0.940	0.998	(0.882,1.127)	0.971	0.800	(0.614,1.042)	0.122	0.942	(0.788,1.126)	0.514	0.915	(0.689,1.215)	0.548	0.888	(0.685,1.151)	0.385
	VEGF	11	0.959	(0.769,1.195)	0.709	1.030	(0.795,1.334)	0.822	0.989	(0.538,1.818)	0.973	0.836	(0.630,1.109)	0.214	0.779	(0.485,1.250)	0.325	0.768	(0.485,1.217)	0.288
**Interleukins**																				
	IL1β	6	1.071	(0.830,1.382)	0.599	1.070	(0.829,1.381)	0.599	0.794	(0.450,1.399)	0.469	1.019	(0.743,1.399)	0.906	1.177	(0.701,1.976)	0.564	0.979	(0.711,1.348)	0.901
	IL1RA	4	0.725	(0.548,0.959)	0.024	0.814	(0.672,0.984)	0.034	0.847	(0.413,1.737)	0.695	0.775	(0.549,1.094)	0.148	0.852	(0.512,1.420)	0.583	0.860	(0.517,1.431)	0.602
	IL2	9	0.987	(0.835,1.165)	0.874	0.974	(0.836,1.134)	0.734	1.108	(0.799,1.537)	0.558	1.047	(0.840,1.304)	0.683	1.043	(0.748,1.453)	0.811	1.062	(0.787,1.433)	0.703
	IL2RA	7	0.924	(0.705,1.211)	0.567	0.960	(0.881,1.046)	0.354	1.156	(0.646,2.067)	0.647	0.989	(0.734,1.332)	0.941	1.044	(0.635,1.714)	0.872	0.981	(0.626,1.537)	0.935
	IL4	9	0.944	(0.723,1.231)	0.669	0.952	(0.756,1.201)	0.679	1.170	(0.678,2.017)	0.591	0.870	(0.608,1.244)	0.446	0.855	(0.507,1.440)	0.572	0.852	(0.499,1.457)	0.575
	IL5	5	0.963	(0.770,1.205)	0.743	1.025	(0.834,1.262)	0.811	0.819	(0.483,1.388)	0.511	0.949	(0.731,1.233)	0.697	0.949	(0.656,1.372)	0.793	0.951	(0.684,1.321)	0.778
	IL6	3	1.024	(0.841,1.248)	0.812	0.889	(0.739,1.067)	0.206	0.763	(0.4212,1.383)	0.537	1.019	(0.827,1.256)	0.858	1.204	(0.905,1.600)	0.330	0.983	(0.781,1.237)	0.896

IL7	11	1.094	(0.959,1.247)	0.181	1.092	(0.964,1.237)	0.167	0.996	(0.707,1.403)	0.980	1.020	(0.860,1.211)	0.817	1.181	(0.862,1.618)	0.325	0.980	(0.813,1.183)	0.840
IL8	4	1.052	(0.706,1.568)	0.803	0.922	(0.708,1.203)	0.551	1.305	(0.373,4.565)	0.717	1.120	(0.784,1.599)	0.534	1.242	(0.689,2.241)	0.523	1.231	(0.705,2.151)	0.518
IL9	6	0.905	(0.717,1.143)	0.401	0.961	(0.785,1.177)	0.700	1.262	(0.725,2.197)	0.457	0.881	(0.647,1.199)	0.420	0.704	(0.429,1.157)	0.225	1.176	(0.753,1.839)	0.508
IL10	11	0.998	(0.845,1.178)	0.978	0.991	(0.842,1.165)	0.909	0.941	(0.663,1.336)	0.741	0.983	(0.797,1.212)	0.870	1.012	(0.690,1.483)	0.954	0.977	(0.794,1.202)	0.831
IL12	12	1.007	(0.864,1.174)	0.929	0.964	(0.844,1.101)	0.584	0.933	(0.712,1.223)	0.627	0.966	(0.821,1.136)	0.677	0.787	(0.550,1.126)	0.217	0.959	(0.817,1.125)	0.617
IL13	15	1.010	(0.895,1.140)	0.873	1.013	(0.909,1.129)	0.812	1.205	(0.903,1.609)	0.227	1.049	(0.885,1.244)	0.581	1.129	(0.859,1.483)	0.400	1.077	(0.873,1.327)	0.500
IL16	9	0.961	(0.813,1.135)	0.637	0.989	(0.838,1.169)	0.901	1.085	(0.831,1.417)	0.566	0.857	(0.690,1.065)	0.163	0.826	(0.579,1.179)	0.323	0.833	(0.597,1.164)	0.316
IL17	9	0.954	(0.731,1.246)	0.732	0.987	(0.787,1.237)	0.910	1.241	(0.752,2.050)	0.427	1.183	(0.858,1.633)	0.305	1.120	(0.641,1.958)	0.701	1.214	(0.824,1.789)	0.356
IL18	17	1.026	(0.901,1.169)	0.695	0.991	(0.883,1.112)	0.880	0.988	(0.766,1.274)	0.927	1.032	(0.872,1.222)	0.713	1.035	(0.771,1.390)	0.822	1.029	(0.771,1.372)	0.849
**others**																			
IFNγ	11	1.023	(0.807,1.296)	0.852	0.977	(0.770,1.240)	0.848	1.138	(0.702,1.847)	0.612	0.952	(0.694,1.306)	0.758	0.864	(0.500,1.494)	0.613	0.917	(0.578,1.456)	0.721
MIF	7	1.080	(0.885,1.317)	0.450	1.050	(0.870,1.267)	0.611	1.039	(0.742,1.454)	0.833	1.043	(0.802,1.356)	0.753	0.983	(0.664,1.456)	0.936	0.986	(0.672,1.446)	0.945
TNFα	4	0.986	(0.643,1.511)	0.947	1.035	(0.736,1.455)	0.843	1.170	(0.427,3.204)	0.789	1.132	(0.800,1.602)	0.483	1.292	(0.763,2.188)	0.410	1.281	(0.807,2.034)	0.370
TNFβ	5	0.937	(0.800,1.097)	0.425	0.959	(0.834,1.102)	0.551	0.866	(0.643,1.167)	0.415	0.895	(0.740,1.083)	0.254	0.874	(0.658,1.161)	0.405	0.854	(0.636,1.146)	0.352
TRAIL	13	1.016	(0.826,1.250)	0.881	1.046	(0.850,1.288)	0.668	1.057	(0.701,1.593)	0.796	1.014	(0.764,1.346)	0.923	1.042	(0.626,1.733)	0.877	0.991	(0.621,1.581)	0.970

Abbreviations: CI, Confidence interval; OR, Odds Ratio; pval, p-value; SNPs, single nucleotide polymorphisms

OR and 95% CI represent the change in the odds ratio of cirrhosis per 1 SD increase in inflammatory cytokines.

After correcting for multiple comparison, p-value < 0.05/41 = 0.0012 was considered as significant.

Supplementary Table S2. Pleiotropy and heterogeneity tests for Dataset 1.

Category	Exposure	Pleiotropy Test				Heterogeneity Test		Recommended Method
		MR Egger Intercept	SE	Pval	PRESSO Pval	Q1 Pval	Q2 Pval	
**Chemokines**	CTACK	-0.113	0.098	0.301	0.117	0.113	0.150	IVW
	Eotaxin	0.061	0.032	0.094	0.355	0.327	0.544	IVW
	GROa	0.029	0.054	0.627	0.795	0.674	0.576	IVW
	IP10	-0.030	0.042	0.609	0.068	0.697	0.634	IVW
	MCP1	0.066	0.041	0.149	0.219	0.167	0.281	IVW
	MCP3	-0.168	0.054	0.090	0.097	0.022	0.997	Weighted Median
	MIG	-0.025	0.033	0.477	0.522	0.532	0.493	IVW
	MIP1α	-0.058	0.063	0.425	0.384	0.554	0.537	IVW
	MIP1β	-0.008	0.014	0.553	0.783	0.551	0.698	IVW
	RANTES	0.035	0.052	0.521	0.446	0.554	0.496	IVW
	SDF1α	0.024	0.031	0.461	0.870	0.886	0.888	IVW
**Growthfactors**	bNGF	-0.091	0.087	0.353	0.594	0.609	0.645	IVW
	FGFBasic	-0.007	0.047	0.891	0.727	0.929	0.855	IVW
	GCSF	-0.028	0.029	0.356	0.675	0.713	0.729	IVW
	HGF	-0.053	0.056	0.381	0.977	0.893	0.918	IVW
	MCSF	0.050	0.041	0.269	0.433	0.476	0.533	IVW
	PDGFbb	-0.014	0.044	0.043	0.184	0.177	0.132	IVW
	SCF	0.013	0.033	0.697	0.909	0.988	0.980	IVW
	SCGFβ	0.052	0.027	0.073	0.534	0.450	0.677	IVW
	VEGF	-0.004	0.038	0.916	0.127	0.394	0.309	IVW
**Interleukins**	IL1β	0.046	0.039	0.311	0.557	0.509	0.568	IVW
	IL1RA	-0.026	0.056	0.690	0.840	0.546	0.384	IVW
	IL2	-0.023	0.029	0.446	0.842	0.868	0.863	IVW
	IL2RA	-0.046	0.054	0.431	0.052	0.072	0.072	IVW
	IL4	-0.037	0.041	0.405	0.887	0.802	0.804	IVW
	IL5	0.033	0.049	0.552	0.675	0.884	0.869	IVW
	IL6	0.062	0.060	0.492	0.473	0.356	0.316	IVW
	IL7	0.025	0.044	0.575	0.940	0.926	0.906	IVW
	IL8	-0.032	0.088	0.750	0.166	0.103	0.055	IVW
	IL9	-0.082	0.064	0.264	0.597	0.493	0.606	IVW
	IL10	0.009	0.023	0.719	0.098	0.847	0.892	IVW
	IL12	0.017	0.025	0.514	0.491	0.239	0.208	IVW
	IL13	-0.038	0.029	0.208	0.556	0.560	0.626	IVW
	IL16	-0.043	0.038	0.292	0.146	0.302	0.331	IVW
	IL17	-0.050	0.042	0.270	0.319	0.213	0.255	IVW
	IL18	0.009	0.026	0.737	0.636	0.894	0.860	IVW
**others**	IFNγ	-0.016	0.032	0.626	0.332	0.392	0.328	IVW
	MIF	0.011	0.038	0.792	0.594	0.570	0.451	IVW
	TNFα	-0.035	0.091	0.736	0.144	0.064	0.034	IVW
	TNFβ	0.029	0.046	0.570	0.297	0.263	0.202	IVW
	TRAIL	-0.006	0.025	0.828	0.312	0.448	0.370	IVW

Q1 pval: p value of Q test from IVW method; Q2 pval: p value of Q test from MR-Egger method
Abbreviations: pval, p-value; Q, Cochran Q statistics; SNPs, single nucleotide polymorphisms; IVW, the inverse variance weighted method.

Supplementary Table S3. SNPs information for Dataset 1.

		Inflammatory cytokines						Cirrhosis		
	SNP	effect allele	other allele	beta	se	pval	F	beta	se	pval
CTACK	rs113031890	G	A	-0.5203	0.112	4.16E-06	21.6	-0.0019	0.1511	0.9901
	rs116943377	A	G	0.2845	0.0613	3.13E-06	21.5	-0.2147	0.0864	0.01293
	rs11790474	T	C	0.1783	0.0312	1.08E-08	32.6	-0.0854	0.0427	0.04565
	rs141885429	A	G	0.3076	0.0674	4.87E-06	20.8	0.0815	0.1033	0.4302
	rs55764737	C	T	-0.5313	0.0972	4.62E-08	29.9	-0.0522	0.1368	0.702999
	rs76395525	A	G	0.5277	0.1083	9.55E-07	23.7	-0.2908	0.1612	0.07128
	rs79810036	A	G	0.3791	0.0822	4.87E-06	21.3	-8.00E-04	0.1028	0.9938
Eotaxin										
	rs11920996	T	C	0.2962	0.0378	5.23E-15	61.4	-0.1412	0.0779	0.07006
	rs1476670	C	A	0.1007	0.0217	3.51E-06	21.5	0.0476	0.0442	0.2814
	rs2040143	A	G	-0.0868	0.0178	1.12E-06	23.8	-0.0041	0.0365	0.9113
	rs2229593	T	C	0.3627	0.0407	6.73E-19	79.4	-0.1082	0.0838	0.1968
	rs2249581	C	T	0.0906	0.018	4.92E-07	25.3	-0.0124	0.0376	0.741199
	rs2419841	C	T	0.1277	0.0279	4.98E-06	20.9	-0.0797	0.0603	0.1867
	rs4683182	G	A	-0.0871	0.0189	4.16E-06	21.2	-0.0239	0.0398	0.5486
	rs5746492	G	A	-0.0954	0.0207	3.96E-06	21.2	0.0275	0.0437	0.5292
	rs73065695	A	G	0.1536	0.0282	5.05E-08	29.7	-0.0744	0.0596	0.212
	rs75426604	A	C	-0.1366	0.0291	2.53E-06	22.0	0.0172	0.0565	0.7602
	rs9317045	C	A	-0.1182	0.0237	5.82E-07	24.9	-0.0808	0.0464	0.08182
GROa										
	rs115214168	T	C	0.4397	0.0831	1.41E-07	28.0	-0.0848	0.1135	0.4552
	rs117078153	C	T	0.2605	0.0573	4.06E-06	20.7	-0.1145	0.0803	0.1541
	rs1361829	G	A	0.1131	0.0243	3.21E-06	21.7	-0.0029	0.0329	0.9293
	rs2422841	A	G	-0.1657	0.0361	4.66E-06	21.1	-0.0261	0.0476	0.5831
	rs3845622	A	C	-0.2407	0.0384	3.51E-10	39.3	-0.019	0.0494	0.7
	rs73020704	G	A	-0.2677	0.0548	1.54E-06	23.8	-0.0343	0.0775	0.6579
IP10										
	rs143799975	G	A	0.7984	0.1637	1.00E-06	23.8	-0.1702	0.1954	0.3837
	rs1951169	G	A	-0.1147	0.0245	2.47E-06	21.9	0.062	0.0341	0.0694
	rs7645625	G	T	0.1086	0.0237	4.41E-06	21.0	-0.0382	0.0333	0.2525
MCP1										
	rs112313229	A	G	-0.1646	0.0313	1.43E-07	27.6	0.0757	0.0639	0.2362
	rs11920996	T	C	0.1811	0.0377	1.63E-06	23.1	-0.1412	0.0779	0.07006
	rs12062235	G	T	-0.1464	0.0321	4.77E-06	20.8	0.0228	0.068	0.737099
	rs12496585	T	G	0.0963	0.021	4.19E-06	21.0	-0.055	0.0428	0.1992
	rs145775149	A	G	-0.5645	0.1239	4.67E-06	20.8	0.2964	0.1889	0.1166
	rs2229593	T	C	0.2655	0.0406	7.54E-11	42.8	-0.1082	0.0838	0.1968
	rs2712431	A	C	-0.0787	0.0172	4.76E-06	20.9	-0.0512	0.0362	0.1573
	rs77116118	C	T	0.4279	0.0827	2.86E-07	26.8	-0.0482	0.1284	0.707301
	rs79939301	A	G	0.1445	0.0256	1.55E-08	31.9	-0.0483	0.052	0.3524
	rs9317045	C	A	-0.1134	0.0236	1.52E-06	23.1	-0.0808	0.0464	0.08182

MCP3										
	rs114615979	T	C	0.6965	0.1511	3.85E-06	21.2	0.2898	0.1063	0.006407
	rs142044459	C	T	0.6925	0.1511	4.35E-06	21.0	0.29	0.1063	0.006373
	rs146167206	T	C	0.7464	0.1534	1.24E-06	23.6	0.3142	0.1162	0.006881
	rs6993671	C	T	-0.2065	0.0447	3.77E-06	21.3	0.0328	0.0337	0.3291
MIG										
	rs10266753	C	T	0.1938	0.04	1.39E-06	23.5	0.032	0.0582	0.5824
	rs111607343	A	G	-0.521	0.1119	2.83E-06	21.7	0.175	0.1429	0.2205
	rs11177248	A	G	0.3073	0.067	4.45E-06	21.0	0.02	0.0844	0.8126
	rs112337562	G	T	0.37	0.0796	2.98E-06	21.6	0.0024	0.1047	0.982
	rs139010077	T	C	0.4322	0.095	3.55E-06	20.7	0.3365	0.1282	0.008668
	rs3733233	C	T	-0.1242	0.0252	8.97E-07	24.3	-0.006	0.0344	0.861
	rs5752128	C	T	0.1685	0.0369	4.34E-06	20.8	0.0428	0.0501	0.3932
	rs62562991	A	G	0.6236	0.126	8.40E-07	24.5	0.1418	0.1376	0.3029
	rs6679677	A	C	0.162	0.0329	8.86E-07	24.2	-0.0271	0.046	0.556
	rs75508933	C	T	-0.7698	0.1658	4.21E-06	21.5	-0.2325	0.2004	0.246
	rs77041705	T	G	0.2534	0.055	4.05E-06	21.2	0.0683	0.0787	0.3853
	rs8127917	T	G	0.2454	0.0493	6.76E-07	24.8	-0.0304	0.0693	0.6605
	rs816960	T	C	-0.1224	0.0244	5.01E-07	25.2	-0.0065	0.034	0.848
MIP1α										
	rs10835056	G	T	-0.1194	0.0254	2.60E-06	22.1	0.0413	0.0348	0.2355
	rs12159394	A	G	-0.1695	0.0368	4.24E-06	21.2	0.0867	0.0511	0.090141
	rs34771762	G	A	-0.249	0.0523	2.13E-06	22.7	-0.0334	0.0639	0.6012
	rs4891392	G	A	-0.2811	0.0607	3.77E-06	21.4	0.0574	0.0826	0.4866
	rs62462206	C	A	0.1212	0.0263	4.24E-06	21.2	-0.0285	0.036	0.4278
MIP1β										
	rs112257251	A	G	-0.1211	0.0196	7.66E-10	38.2	0.0929	0.0415	0.02506
	rs116237296	A	G	0.5437	0.1115	7.23E-07	23.8	-0.0174	0.2456	0.9435
	rs1437220	C	T	-0.1478	0.0315	3.53E-06	22.0	-0.0201	0.0611	0.742499
	rs145526037	G	T	0.2003	0.0407	8.66E-07	24.2	0.0654	0.0844	0.4381
	rs17138331	G	A	0.1391	0.0295	2.26E-06	22.2	0.0144	0.0593	0.8082
	rs281730	A	C	0.0781	0.0171	4.90E-06	20.9	0.0036	0.0355	0.9197
	rs4683034	G	A	-0.0729	0.0157	3.47E-06	21.6	0.0341	0.033	0.3016
	rs57934583	A	G	-0.1201	0.0183	4.90E-11	43.1	-0.0062	0.0378	0.8689
	rs6441959	G	A	0.1291	0.0219	3.08E-09	34.7	0.0465	0.0463	0.3154
	rs72791296	T	C	0.2369	0.0466	3.78E-07	25.8	-0.0629	0.0924	0.4961
	rs72799710	T	C	-0.1014	0.0218	3.21E-06	21.6	0.0246	0.0453	0.5872
	rs72820259	A	G	-0.3305	0.0506	7.57E-11	42.7	-0.1938	0.1025	0.05879
	rs74810984	C	T	-0.2206	0.0474	1.96E-06	21.7	0.0254	0.1029	0.8053
	rs75192081	A	G	0.1273	0.0195	6.59E-11	42.6	-0.0331	0.041	0.4191
	rs76582507	A	G	0.3175	0.0677	3.26E-06	22.0	0.2363	0.1865	0.2052
	rs76583883	T	G	-0.2317	0.0511	4.99E-06	20.6	0.0207	0.0897	0.8172
	rs9903158	C	T	0.1671	0.0293	1.16E-08	32.5	-0.0143	0.0615	0.8156
RANTES										

	rs10505135	C	T	-0.1318	0.0253	1.86E-07	27.1	-0.0217	0.0341	0.5253
	rs117640470	T	C	0.2892	0.0602	1.50E-06	23.1	-0.0207	0.0835	0.8043
	rs148526102	T	C	-0.3832	0.0833	4.36E-06	21.1	0.0862	0.1112	0.4385
	rs4940620	G	A	0.2494	0.054	3.54E-06	21.3	0.0457	0.0741	0.5372
	rs62438851	G	A	0.1957	0.0414	2.33E-06	22.3	-0.1204	0.0543	0.0266
	rs72793342	A	G	-0.1487	0.0308	1.48E-06	23.3	-0.0024	0.0404	0.9522
	rs75613039	T	C	0.37	0.081	4.81E-06	20.9	3.00E-04	0.1011	0.9976
	rs78050316	A	C	0.428	0.0862	6.71E-07	24.6	-0.0818	0.1249	0.512201
	rs818450	T	C	0.2345	0.0504	3.34E-06	21.6	-0.0862	0.0677	0.2026
SDF1α										
	rs13106842	C	T	-0.0948	0.0208	4.08E-06	20.8	-0.0133	0.0404	0.742299
	rs13400104	G	A	0.0647	0.0189	4.53E-06	11.7	0.0422	0.0366	0.2489
	rs139840550	A	G	0.1834	0.0549	3.79E-06	11.2	0.0911	0.1191	0.4442
	rs144494443	G	A	-0.4595	0.1	4.82E-06	21.1	0.0827	0.1975	0.675601
	rs149893336	G	A	0.5034	0.1081	4.52E-06	21.7	-0.0641	0.1553	0.68
	rs4581824	G	T	0.0701	0.0173	3.05E-06	16.4	-0.0044	0.0344	0.8974
	rs67689854	A	C	-0.0681	0.0195	3.07E-06	12.2	0.0031	0.0404	0.9384
bNGF										
	rs28637706	T	G	-0.1589	0.0263	1.42E-09	36.5	-0.062	0.0356	0.081261
	rs67476890	T	C	0.1769	0.0379	3.13E-06	21.8	0.0245	0.0523	0.639401
	rs71641308	T	C	0.2043	0.0432	2.30E-06	22.4	0.0309	0.0558	0.5801
	rs72780728	A	G	0.1883	0.0403	2.99E-06	21.8	0.0116	0.0563	0.8368
	rs7970581	G	T	-0.138	0.0282	9.27E-07	23.9	0.0256	0.0369	0.488
	rs9436119	A	G	-0.1121	0.0246	3.91E-06	20.8	0.0168	0.0332	0.6134
FGFBasic										
	rs13412535	A	G	-0.1112	0.0225	7.35E-07	24.4	0.013	0.0414	0.752501
	rs147409637	T	C	0.2049	0.0432	1.79E-06	22.5	-0.1128	0.088	0.1998
	rs2118824	G	A	-0.0751	0.0164	4.55E-06	21.0	0.0363	0.0328	0.2676
	rs2849358	A	G	0.0898	0.0193	3.31E-06	21.6	-0.0044	0.0378	0.9072
	rs4795091	G	A	-0.1273	0.0267	1.49E-06	22.7	0.0199	0.0529	0.7076
	rs78523761	A	G	0.4758	0.1032	4.33E-06	21.3	-0.027	0.2075	0.8966
GCSF										
	rs10939033	A	G	-0.0764	0.0164	3.04E-06	21.7	-0.016	0.0335	0.632199
	rs147128865	T	C	0.27	0.0587	4.92E-06	21.2	0.1193	0.1106	0.2805
	rs1817411	T	C	0.089	0.0191	3.10E-06	21.7	-0.033	0.0398	0.4074
	rs183023730	T	G	0.7952	0.1677	2.06E-06	22.5	0.3304	0.2655	0.2132
	rs6740648	C	T	-0.0799	0.0172	3.20E-06	21.6	0.0437	0.0351	0.2128
	rs7253856	T	C	0.0917	0.0195	2.42E-06	22.1	0.0147	0.0396	0.7093
	rs74148555	T	C	-0.3715	0.0755	1.55E-06	24.2	0.0456	0.116	0.6941
	rs77318030	C	T	0.2045	0.0428	2.21E-06	22.8	0.0075	0.0814	0.927
	rs78523761	A	G	0.5435	0.1139	1.84E-06	22.8	-0.027	0.2075	0.8966
HGF										
	rs150322232	G	A	-0.2104	0.0463	4.89E-06	20.6	-0.062	0.0854	0.467599
	rs16844364	A	G	0.0954	0.0203	2.50E-06	22.1	-0.0314	0.0427	0.4621

	rs1756279	T	C	0.17	0.0374	4.39E-06	20.7	0.0229	0.0782	0.7701
	rs1800493	T	C	0.3557	0.077	3.57E-06	21.3	0.095	0.1463	0.516
	rs2003620	T	C	0.2279	0.0489	2.83E-06	21.7	0.1558	0.1001	0.1197
	rs5745687	T	C	-0.3072	0.0406	2.75E-14	57.2	-0.0268	0.0824	0.745501
	rs62481625	C	T	-0.1091	0.0225	1.18E-06	23.5	-0.0086	0.0458	0.8514
	rs80051150	C	T	-0.197	0.0414	1.97E-06	22.6	-0.0357	0.0894	0.6892
MCSF										
	rs116274860	G	T	-0.819	0.1741	2.74E-06	22.1	0.26	0.1479	0.07887
	rs117867915	C	T	-0.5272	0.1098	1.61E-06	23.0	0.0887	0.1172	0.4488
	rs12962919	T	C	0.3052	0.0662	4.65E-06	21.2	0.0923	0.0649	0.1547
	rs145778765	T	C	-0.7993	0.1689	2.20E-06	22.3	0.0327	0.119	0.7833
	rs147378920	G	A	0.6109	0.1318	2.93E-06	21.4	0.0834	0.115	0.4683
	rs56041615	T	G	-0.2565	0.0563	4.87E-06	20.7	-0.0107	0.0649	0.8686
	rs62294910	A	G	0.3431	0.0691	6.82E-07	24.6	-0.0041	0.0677	0.9522
	rs9387100	C	T	0.1352	0.0292	4.07E-06	21.4	0.017	0.033	0.606599
PDGFbb										
	rs10512952	T	C	-0.2879	0.0588	5.54E-07	24.0	-0.3289	0.1211	0.006621
	rs11766649	G	A	-0.0908	0.0196	3.53E-06	21.5	0.0187	0.0406	0.6453
	rs11916118	G	A	-0.0889	0.0194	4.93E-06	21.0	-0.0234	0.0413	0.5703
	rs12289510	G	A	0.078	0.0158	7.69E-07	24.4	-0.0198	0.0329	0.5469
	rs12615784	C	T	0.1021	0.0193	1.18E-07	28.0	0.0698	0.04	0.08067
	rs147862316	T	C	0.232	0.0411	1.64E-08	31.9	0.0573	0.0896	0.5224
	rs2643354	A	G	0.124	0.0261	2.09E-06	22.6	0.0611	0.0552	0.2685
	rs35859699	A	G	-0.3952	0.0842	2.07E-06	22.0	-0.0132	0.1558	0.9325
	rs62191444	T	G	-0.1115	0.0239	3.02E-06	21.8	0.0435	0.0459	0.3438
	rs6910518	G	T	-0.0812	0.0162	5.60E-07	25.1	-0.0604	0.0337	0.07283
	rs73162807	A	C	-0.2391	0.0499	1.74E-06	23.0	0.0612	0.1066	0.5661
SCF										
	rs10491875	T	C	-0.076	0.0166	4.19E-06	21.0	0.0027	0.0342	0.9362
	rs10800449	A	C	0.0874	0.0179	1.37E-06	23.8	-0.0057	0.0363	0.8744
	rs11244035	T	C	-0.1297	0.028	4.01E-06	21.5	0.0292	0.0586	0.6178
	rs113127926	A	C	0.1982	0.042	2.27E-06	22.3	-0.0379	0.0841	0.6522
	rs11847575	A	G	-0.1029	0.0226	4.60E-06	20.7	0.0232	0.0441	0.5987
	rs13412535	A	G	-0.1067	0.0213	6.04E-07	25.1	0.013	0.0414	0.752501
	rs138538809	T	C	-0.5747	0.1139	3.18E-07	25.5	-0.0165	0.2267	0.942
	rs1536480	T	C	0.0804	0.0168	1.64E-06	22.9	0.041	0.0349	0.2397
	rs1942355	T	C	-0.0716	0.0157	4.70E-06	20.8	-0.0034	0.0328	0.9185
	rs78666213	G	T	0.2744	0.0576	2.59E-06	22.7	-0.0082	0.114	0.9427
SCGFβ										
	rs10800449	A	C	0.1431	0.0266	1.24E-07	28.9	-0.0057	0.0363	0.8744
	rs112346514	T	C	-0.3314	0.0711	2.37E-06	21.7	0.0479	0.0852	0.5737
	rs118003677	C	T	0.368	0.0786	2.81E-06	21.9	-0.1793	0.11	0.103
	rs12480722	C	T	-0.1624	0.0355	4.72E-06	20.9	0.0129	0.0489	0.7915
	rs13866	T	C	-0.165	0.0281	3.66E-09	34.5	-0.0316	0.0375	0.3995

	rs139413256	A	G	-0.5377	0.1084	7.04E-07	24.6	-0.0656	0.1322	0.619901
	rs143829871	C	T	0.1902	0.04	1.90E-06	22.6	0.017	0.0565	0.7637
	rs144724875	T	C	0.5459	0.084	9.19E-11	42.2	-0.0871	0.0981	0.375
	rs151194174	A	G	0.4635	0.0942	1.13E-06	24.2	-0.0661	0.0856	0.4399
	rs264157	G	A	-0.1091	0.0234	3.13E-06	21.7	0.0159	0.0328	0.6264
	rs34911860	A	G	-0.3675	0.0789	3.24E-06	21.7	-0.0404	0.1257	0.747699
	rs3817303	T	G	0.1389	0.0295	3.17E-06	22.2	0.0881	0.0415	0.03399
	rs4737731	T	C	0.1144	0.0252	4.95E-06	20.6	0.0683	0.0362	0.05884
	rs7762066	C	T	-0.1389	0.0299	3.50E-06	21.6	-0.0095	0.0388	0.8061
	rs78217154	C	T	-0.3997	0.0864	3.77E-06	21.4	0.0217	0.1175	0.8535
VEGF										
	rs10153304	A	G	0.1547	0.0325	1.94E-06	22.7	0.1156	0.0595	0.05202
	rs10822118	C	T	0.0786	0.0169	3.14E-06	21.6	0.0413	0.0328	0.2074
	rs10967183	T	C	-0.0887	0.017	1.72E-07	27.2	-0.0076	0.0331	0.8175
	rs111290542	C	T	-0.0955	0.0201	2.17E-06	22.6	0.031	0.0338	0.3592
	rs144566792	T	C	-0.3036	0.0668	3.96E-06	20.7	0.076	0.0961	0.429
	rs56071907	T	C	0.1255	0.0271	3.92E-06	21.4	-0.0181	0.0472	0.7011
	rs60013354	G	A	0.2491	0.0521	1.74E-06	22.9	0.0552	0.0989	0.577
	rs6496613	C	A	0.2419	0.0516	3.41E-06	22.0	-0.0495	0.1002	0.6215
	rs77961527	G	A	-0.2242	0.0459	1.90E-06	23.9	-0.027	0.0771	0.726001
	rs7867894	C	T	-0.1038	0.0194	9.01E-08	28.6	0.0371	0.0373	0.3208
	rs844294	C	T	-0.1059	0.0171	5.30E-10	38.3	0.0467	0.033	0.1576
IL1β										
	rs143319329	T	C	0.2801	0.0715	2.00E-06	15.3	0.3181	0.2043	0.1194
	rs1942793	T	G	0.0717	0.0187	4.98E-06	14.7	0.035	0.0327	0.2848
	rs4786740	A	C	0.0845	0.0202	4.67E-06	17.5	0.0224	0.0335	0.5038
	rs61335305	A	C	0.2966	0.0724	1.90E-06	16.8	-0.0593	0.124	0.6326
	rs62015704	G	A	-0.1082	0.0283	2.09E-06	14.6	-0.0225	0.0496	0.6507
	rs9898641	C	T	0.2032	0.0454	3.59E-06	20.0	-0.0113	0.0341	0.740399
IL1RA										
	rs1054402	C	T	-0.1311	0.027	1.13E-06	23.6	0.0146	0.0378	0.699199
	rs56134659	G	A	0.1117	0.0237	2.44E-06	22.2	-0.0509	0.0327	0.1198
	rs56410028	G	A	0.179	0.039	4.46E-06	21.1	-0.1066	0.0509	0.03609
	rs61335305	A	C	0.4453	0.0908	1.00E-06	24.0	-0.0593	0.124	0.6326
IL2										
	rs12051139	C	T	0.1131	0.0247	4.76E-06	21.0	-0.0066	0.0334	0.8439
	rs13412535	A	G	0.1764	0.0332	1.18E-07	28.2	0.013	0.0414	0.752501
	rs170117	T	C	-0.1617	0.0349	3.87E-06	21.5	-0.0372	0.0467	0.4255
	rs2807544	G	A	-0.1175	0.0253	3.41E-06	21.6	0.0207	0.0339	0.542
	rs4634519	G	A	0.1261	0.0269	2.77E-06	22.0	-0.0548	0.0367	0.1354
	rs61335305	A	C	0.4514	0.0918	7.32E-07	24.2	-0.0593	0.124	0.6326
	rs62124990	T	G	-0.6961	0.1495	3.22E-06	21.7	-0.0502	0.1213	0.6789
	rs7615304	G	A	0.1172	0.0242	1.21E-06	23.4	-5.00E-04	0.0329	0.989
	rs80336398	C	T	-0.4001	0.0858	2.82E-06	21.7	-0.0381	0.1052	0.716901

IL2RA										
	rs10110247	A	G	-0.1337	0.0293	4.53E-06	20.8	-0.037	0.0404	0.3601
	rs10490280	C	T	-0.1396	0.0304	3.59E-06	21.1	0.0022	0.042	0.9585
	rs117244812	A	G	-0.7064	0.1488	2.10E-06	22.5	-0.334	0.1968	0.0897
	rs34037190	A	G	0.4679	0.094	6.02E-07	24.8	0.0336	0.1018	0.7413
	rs6881097	G	A	0.1145	0.0251	4.83E-06	20.8	-0.0576	0.0351	0.1004
	rs7078614	T	G	-0.1555	0.0242	1.36E-10	41.3	0.0394	0.0341	0.2484
	rs7678976	A	G	0.3296	0.0715	4.04E-06	21.2	-0.1828	0.0876	0.03685
IL4										
	rs10512267	C	T	0.0824	0.0161	2.94E-07	26.2	-0.0165	0.0332	0.6181
	rs116705532	G	T	0.4678	0.0978	1.76E-06	22.9	-0.0789	0.2037	0.6984
	rs117146485	C	T	0.2924	0.0629	2.71E-06	21.6	-0.0658	0.1232	0.5932
	rs17713451	A	G	0.1274	0.0253	4.97E-07	25.4	-0.007	0.0519	0.8932
	rs73023729	A	G	-0.1796	0.0366	9.03E-07	24.1	0.026	0.0795	0.7431
	rs7613691	G	A	-0.1775	0.0384	4.05E-06	21.4	0.016	0.0758	0.8324
	rs79597994	T	C	-0.5831	0.127	4.32E-06	21.1	-0.1636	0.1982	0.409
	rs9508291	C	T	0.1676	0.0359	3.03E-06	21.8	0.0944	0.0756	0.212
	rs9941733	G	A	-0.114	0.0229	6.88E-07	24.8	0.0558	0.0433	0.198
IL5										
	rs11680908	G	A	-0.2634	0.0554	2.03E-06	22.6	0.0179	0.0746	0.8103
	rs6737109	C	T	-0.116	0.0247	2.40E-06	22.0	-0.0239	0.0329	0.467599
	rs72831687	A	G	-0.5239	0.1109	1.69E-06	22.3	0.0181	0.1288	0.8881
	rs73040130	C	T	-0.2638	0.0529	6.00E-07	24.9	0.0558	0.0717	0.4364
	rs7767396	G	A	-0.1515	0.0246	7.69E-10	37.9	0.0081	0.0328	0.8055
IL6										
	rs12028290	A	C	-0.3289	0.03777	2.61E-06	22.1	0.130129	0.280695	0.642936
	rs12408012	G	A	-0.1164	0.2256	7.34E-08	29.3	-0.08184	0.123204	0.506533
	rs16840102	A	G	0.2672	0.0338	9.58E-07	23.3	-0.18423	0.291292	0.527087
	rs17344940	T	G	0.0738	0.4264	3.17E-06	21.8	-0.04325	0.105164	0.680889
	rs2455137	T	C	0.2692	0.08449	2.43E-06	22.2	0.03064	0.219673	0.889072
	rs2926482	G	C	-0.1786	0.162	3.74E-06	21.2	-0.06851	0.133689	0.608321
	rs7364349	A	G	-0.1311	0.2664	1.13E-06	23.6	-0.17586	0.121268	0.147013
IL7										
	rs117509142	C	T	0.327	0.0688	1.99E-06	22.6	-0.0219	0.0911	0.8099
	rs142397827	A	C	0.4555	0.0993	4.50E-06	21.0	-0.0214	0.1171	0.8549
	rs144701438	A	G	-0.4819	0.0989	9.75E-07	23.7	-0.0645	0.1374	0.6385
	rs17091524	C	T	-0.4924	0.1013	1.91E-06	23.6	-0.1033	0.1256	0.4108
	rs28793375	T	C	0.1638	0.0361	4.46E-06	20.6	0.0381	0.048	0.4269
	rs62006410	T	C	-0.1557	0.0303	3.39E-07	26.4	-0.0505	0.0383	0.1876
	rs75697425	A	C	0.1632	0.0346	2.54E-06	22.2	0.0229	0.0461	0.6192
	rs77318030	C	T	0.2921	0.0632	3.74E-06	21.3	0.0075	0.0814	0.927
	rs7739450	A	G	-0.2961	0.0254	1.07E-30	135.8	0.008	0.0331	0.8098
	rs77981494	C	T	0.5178	0.1064	1.07E-06	23.7	0.1771	0.1191	0.1372
	rs9296421	G	T	-0.169	0.0355	2.10E-06	22.6	-0.0249	0.0437	0.569399

IL8										
	rs11634944	C	T	0.1214	0.0252	1.29E-06	23.2	-0.0596	0.0345	0.08437
	rs12075	A	G	0.12	0.0236	3.88E-07	25.8	0.0547	0.0328	0.09559
	rs141926526	C	A	0.6149	0.1308	2.57E-06	22.1	0.1369	0.1838	0.4563
	rs2673604	A	C	-0.1266	0.0255	7.02E-07	24.6	-1.00E-04	0.0353	0.9978
IL9										
	rs1259728	A	G	-0.2402	0.0508	3.10E-06	22.3	0.0848	0.0708	0.2308
	rs41294750	T	C	0.3514	0.0748	2.37E-06	22.1	0.0405	0.0984	0.680899
	rs4880409	T	C	-0.3355	0.0723	3.50E-06	21.5	0.1532	0.1643	0.3511
	rs61867538	T	C	0.3566	0.0774	3.93E-06	21.2	0.0686	0.0803	0.3929
	rs7232268	G	A	-0.2759	0.0587	2.53E-06	22.1	0.0588	0.0831	0.4792
	rs7242404	A	G	-0.1228	0.0264	3.27E-06	21.6	0.043	0.0363	0.2365
IL10										
	rs10457128	A	G	-0.0865	0.0172	5.24E-07	25.3	-0.0472	0.0342	0.1673
	rs10493718	A	C	-0.11	0.0222	7.16E-07	24.5	-0.0166	0.0451	0.713001
	rs11206302	T	C	-0.1189	0.0251	2.20E-06	22.4	-0.0036	0.0499	0.9433
	rs2086656	T	C	-0.0789	0.0171	3.78E-06	21.3	0.0141	0.0344	0.6819
	rs282258	C	T	-0.0992	0.0162	1.00E-09	37.5	0.0315	0.0331	0.3404
	rs3025021	C	T	-0.0947	0.0195	1.46E-06	23.6	0.0168	0.0349	0.6306
	rs4349809	G	T	-0.2853	0.0165	5.77E-67	298.9	0.0066	0.0329	0.8401
	rs465757	A	G	0.084	0.0174	1.17E-06	23.3	0.0337	0.0344	0.328
	rs6085948	A	G	0.098	0.0202	1.25E-06	23.5	0.01	0.0402	0.8041
	rs6458375	T	C	0.0946	0.0198	2.07E-06	22.8	-0.0279	0.0391	0.4752
	rs7088799	G	T	0.0852	0.0167	3.23E-07	26.0	0.009	0.0337	0.7904
IL12										
	rs12199215	T	C	0.1278	0.0192	5.11E-11	44.3	0.0787	0.0386	0.04168
	rs145023524	A	G	0.279	0.0394	1.54E-12	50.1	-0.0759	0.0827	0.3584
	rs17229494	G	A	0.1172	0.0257	4.93E-06	20.8	0.1026	0.0523	0.04986
	rs282258	C	T	-0.073	0.0156	3.21E-06	21.9	0.0315	0.0331	0.3404
	rs3025021	C	T	-0.0898	0.0188	2.20E-06	22.8	0.0168	0.0349	0.6306
	rs4349809	G	T	-0.3777	0.0159	2.56E-124	564.2	0.0066	0.0329	0.8401
	rs71361173	G	T	-0.111	0.0239	3.06E-06	21.6	-0.0445	0.0494	0.3675
	rs72831623	A	G	0.1913	0.037	2.42E-07	26.7	-0.0425	0.0656	0.517299
	rs7754905	G	A	0.1029	0.019	4.28E-08	29.3	-0.0279	0.0392	0.4775
	rs7757246	C	T	-0.139	0.0285	1.97E-06	23.8	0.0499	0.0587	0.3955
	rs782107	A	G	0.075	0.0156	1.60E-06	23.1	0.039	0.0328	0.2339
	rs79121401	C	T	-0.5548	0.1206	4.24E-06	21.2	-0.0057	0.2469	0.9817
IL13										
	rs117795020	A	G	-0.3522	0.0716	9.86E-07	24.2	-0.1179	0.0974	0.2258
	rs12524926	T	C	-0.1623	0.0302	9.53E-08	28.9	-0.0226	0.0405	0.5779
	rs12623722	A	G	-0.1185	0.0258	4.19E-06	21.1	-0.0294	0.0356	0.4082
	rs13209117	A	G	0.1412	0.0286	6.98E-07	24.4	-0.0294	0.0379	0.4389
	rs138854806	A	G	-0.4256	0.0845	6.47E-07	25.4	0.018	0.0977	0.8537
	rs139083458	T	C	0.9902	0.2107	2.81E-06	22.1	0.1685	0.283	0.551501

	rs27949	T	C	-0.1168	0.0252	3.43E-06	21.5	0.0801	0.035	0.02216
	rs28442067	G	A	0.1361	0.0288	2.24E-06	22.3	0.0327	0.0399	0.4122
	rs7073807	C	T	-0.1682	0.0356	2.37E-06	22.3	-0.0092	0.048	0.8477
	rs7747448	A	G	-0.1425	0.028	4.57E-07	25.9	0.0508	0.0376	0.1771
	rs7757246	C	T	-0.2171	0.0423	3.53E-07	26.3	0.0499	0.0587	0.3955
	rs7764227	T	G	-0.2904	0.0261	1.34E-28	123.7	-0.0088	0.036	0.8072
	rs77955971	A	C	0.4309	0.0869	3.50E-06	24.6	0.0357	0.0903	0.692901
	rs9296421	G	T	-0.1746	0.0349	6.30E-07	25.0	-0.0249	0.0437	0.569399
	rs9381249	C	T	0.2688	0.0574	2.84E-06	21.9	0.0274	0.0807	0.7347
IL16										
	rs117217798	T	C	-0.2036	0.0444	4.15E-06	21.0	-0.048	0.059	0.4166
	rs117638452	A	G	0.3911	0.087	4.24E-06	20.2	-0.0601	0.0918	0.5131
	rs12577604	C	T	-0.4615	0.0945	1.01E-06	23.8	0.1157	0.1255	0.3565
	rs12765671	A	G	-0.6023	0.1318	4.84E-06	20.9	-0.0381	0.1827	0.8349
	rs142332135	A	G	-0.7581	0.1083	5.85E-13	49.0	0.1524	0.1274	0.2317
	rs144691581	A	G	0.4882	0.0967	4.20E-07	25.5	-0.0033	0.1119	0.9766
	rs35834666	C	T	0.1664	0.0349	1.58E-06	22.7	-0.0359	0.0464	0.4391
	rs4778640	G	A	-0.7216	0.0988	2.76E-13	53.3	-0.2568	0.131	0.05001
	rs7097884	T	C	-0.112	0.0244	4.30E-06	21.1	0.0457	0.0331	0.1672
IL17										
	rs117029961	A	G	0.4585	0.1015	4.94E-06	20.4	0.043	0.1901	0.8211
	rs117556572	T	C	-0.5102	0.1099	3.28E-06	21.5	-0.1333	0.1095	0.2236
	rs1530455	C	T	-0.108	0.0173	4.87E-10	39.0	-0.0217	0.0342	0.5266
	rs17106604	T	C	0.1129	0.0225	6.37E-07	25.2	-0.0162	0.0461	0.7252
	rs17282552	C	T	0.2001	0.0405	8.21E-07	24.4	0.0544	0.0742	0.4632
	rs187475560	T	C	-0.2434	0.052	3.29E-06	21.9	0.1739	0.0981	0.07622
	rs62191444	T	G	-0.1136	0.0247	4.22E-06	21.1	0.0435	0.0459	0.3438
	rs78296352	T	G	0.3027	0.0646	4.27E-06	22.0	-0.0904	0.1408	0.5208
	rs78612928	C	T	-0.1037	0.0222	2.62E-06	21.8	0.0879	0.0444	0.04771
IL18										
	rs10238937	G	A	-0.3036	0.0661	4.38E-06	21.1	-0.0338	0.0892	0.704399
	rs10409850	G	A	-0.1749	0.035	5.73E-07	25.0	-0.0083	0.0484	0.8632
	rs115763380	A	G	0.3497	0.0751	2.70E-06	21.7	0.0377	0.1058	0.721499
	rs117266781	T	C	0.6841	0.1468	3.15E-06	21.7	0.0221	0.1781	0.9012
	rs11951414	T	C	0.1225	0.0258	2.11E-06	22.5	-0.0214	0.0361	0.5538
	rs12419156	C	T	-0.1665	0.0337	7.53E-07	24.4	-0.0493	0.0423	0.2443
	rs139468359	C	T	-0.5067	0.1099	4.02E-06	21.2	0.1867	0.1482	0.2077
	rs139727649	C	T	0.3698	0.0754	9.37E-07	24.0	0.1852	0.1175	0.115
	rs144841621	T	C	0.518	0.1141	3.81E-06	20.6	-0.009	0.1675	0.9574
	rs1540074	C	T	-0.1098	0.024	4.99E-06	20.9	-0.0326	0.0337	0.334
	rs1979967	T	C	0.1402	0.0286	9.45E-07	24.0	-0.0333	0.0398	0.4025
	rs2729385	A	G	0.1231	0.0262	3.79E-06	22.1	0.0021	0.0365	0.9538
	rs5744267	C	T	-0.268	0.0469	1.33E-08	32.6	-0.0128	0.0575	0.8236
	rs62312914	T	C	-0.1309	0.0251	1.97E-07	27.2	-0.0029	0.0339	0.9325

	rs77187209	C	T	0.4838	0.1046	4.33E-06	21.4	0.1277	0.1381	0.3553
	rs78623212	T	C	0.8705	0.1778	6.71E-07	24.0	-0.1408	0.1882	0.4544
	rs78716465	A	G	0.3265	0.0682	1.63E-06	22.9	-0.0545	0.0887	0.538801
IFNγ										
	rs10733789	C	T	0.0823	0.0177	3.78E-06	21.6	0.0275	0.0358	0.4434
	rs113399544	A	G	-0.0865	0.0183	2.36E-06	22.3	0.0183	0.0363	0.6144
	rs113600793	A	C	0.1829	0.0373	8.95E-07	24.0	-0.0462	0.0706	0.5128
	rs115729819	G	A	-0.2484	0.0515	1.38E-06	23.3	-0.1002	0.1015	0.3234
	rs117046255	T	C	-0.0946	0.0207	4.82E-06	20.9	0.07	0.0422	0.096741
	rs11843756	G	T	-0.184	0.0393	3.09E-06	21.9	-0.1511	0.0806	0.060811
	rs12420286	C	T	-0.2376	0.0501	2.08E-06	22.5	0.09	0.0998	0.367
	rs147378920	G	A	0.3863	0.0754	3.45E-07	26.2	0.0834	0.115	0.4683
	rs1867282	T	C	0.0774	0.0166	3.15E-06	21.7	-0.0124	0.0333	0.709099
	rs2073438	A	G	0.0898	0.0188	1.68E-06	22.8	0.0254	0.0375	0.4983
	rs74148555	T	C	-0.3732	0.0774	2.64E-06	23.2	0.0456	0.116	0.6941
MIF										
	rs1007888	T	C	-0.1265	0.0246	2.73E-07	26.4	-0.0052	0.0337	0.8782
	rs113218956	A	G	-0.8948	0.1879	2.26E-06	22.7	0.0412	0.2588	0.8736
	rs118055855	C	T	-0.6907	0.15	4.13E-06	21.2	0.0613	0.184	0.738999
	rs12594190	G	A	-0.1355	0.0267	3.70E-07	25.7	-0.0102	0.0359	0.776401
	rs141009259	C	T	0.6178	0.1322	2.47E-06	21.8	0.1961	0.1571	0.2121
	rs35792361	A	G	-0.2579	0.0528	1.72E-06	23.8	-0.117	0.0704	0.096321
	rs78098071	C	T	0.4867	0.0918	1.78E-07	28.1	-0.1161	0.1292	0.3687
TNFα										
	rs11029571	G	A	0.1212	0.0262	3.71E-06	21.4	0.039	0.0362	0.2811
	rs116736594	T	C	0.348	0.0711	1.12E-06	23.9	0.0978	0.0904	0.2792
	rs79105320	A	G	0.5605	0.1179	3.59E-06	22.6	-0.0424	0.1541	0.783
	rs912086	A	G	0.1298	0.028	3.59E-06	21.5	-0.084	0.0381	0.02737
TNFβ										
	rs10925040	T	C	0.1755	0.0373	2.67E-06	22.1	0.0399	0.0341	0.2421
	rs143259067	C	T	0.7156	0.1004	5.49E-13	50.7	-0.1683	0.0881	0.05612
	rs2420873	G	T	-0.1682	0.0365	4.12E-06	21.2	0.0254	0.0336	0.4488
	rs62284710	G	A	-0.3625	0.0782	4.36E-06	21.5	0.0382	0.0697	0.583399
	rs76225863	A	G	0.7742	0.123	1.08E-10	39.6	0.045	0.1042	0.6654
TRAIL										
	rs113057689	A	G	-0.2655	0.0491	8.53E-08	29.2	-0.075	0.0788	0.3411
	rs11618126	G	A	-0.8908	0.1914	1.46E-06	21.7	0.1174	0.3868	0.7615
	rs11657269	G	A	-0.1188	0.026	4.78E-06	20.9	0.0013	0.0539	0.9805
	rs11699445	G	T	-0.0746	0.0161	3.27E-06	21.5	-0.0385	0.033	0.243
	rs12492800	C	A	0.0763	0.0158	1.46E-06	23.3	-0.0024	0.0329	0.9425
	rs13115587	A	C	0.1019	0.0218	3.10E-06	21.8	0.0146	0.0464	0.752899
	rs13185784	A	G	0.0846	0.0183	3.90E-06	21.4	-0.0484	0.0374	0.196
	rs13278062	T	G	0.0801	0.0157	3.57E-07	26.0	0.0034	0.0329	0.9165
	rs1357804	T	C	0.1011	0.0207	1.13E-06	23.8	0.0708	0.0436	0.1042

rs146783010	G	A	0.6016	0.135	4.83E-06	19.9	0.2596	0.2402	0.2799
rs747324	C	T	0.0855	0.0178	1.61E-06	23.1	-0.061	0.0365	0.094569
rs75928541	A	G	0.275	0.0593	4.24E-06	21.5	0.0262	0.1116	0.8146
rs9963048	C	T	0.2756	0.0306	2.04E-19	81.1	-0.0701	0.0644	0.2761

Abbreviations: log(OR): log odds ratio; se: standard error a Outliers detected by MR-PRESSO were removed. Beta for inflammatory cytokines represent change in standard deviation per 1 copy of effect allele.

Supplementary Table S4. The reverse MR estimates for Dataset 1.

**Category**	**Exposure**	**No. of SNPs**	**IVW**			**MR-Lasso**			**MR-Egger**			**Weighted Median**			**Simple Mode**			**Weighted Mode**		
			**OR**	**95% CI**	**pval**	**OR**	**95% CI**	**pval**	**OR**	**95% CI**	**pval**	**OR**	**95% CI**	**pval**	**OR**	**95% CI**	**pval**	**OR**	**95% CI**	**pval**
**Chemokines**																				
	CTACK	6	1.024	(0.963-1.089)	0.455	1.031	(0.963,1.089)	0.455	1.029	(0.741-1.429)	0.873	1.031	(0.957-1.110)	0.421	1.032	(0.936-1.138)	0.558	1.032	(0.9441-1.127)	0.521
	Eotaxin	6	1.033	(0.991-1.077)	0.125	1.021	(0.991,1.077)	0.125	0.865	(0.689-1.086)	0.279	1.014	(0.965-1.066)	0.582	1.015	(0.958-1.074)	0.641	1.014	(0.954-1.078)	0.668
	GROa	6	0.951	(0.893-1.013)	0.117	1.033	(0.893,1.013)	0.117	0.759	(0.543-1.061)	0.182	0.937	(0.870-1.009)	0.087	0.936	(0.853-1.026)	0.215	0.936	(0.856-1.023)	0.202
	IP10	6	1.019	(0.957-1.083)	0.542	1.031	(0.959,1.083)	0.542	1.066	(0.769-1.480)	0.720	1.019	(0.944-1.099)	0.629	1.001	(0.909-1.103)	0.978	1.017	(0.933-1.108)	0.723
	MCP1	6	0.967	(0.9283-1.008)	0.115	1.021	(0.929,1.008)	0.115	0.927	(0.740-1.161)	0.546	0.963	(0.915-1.013)	0.140	0.963	(0.902-1.027)	0.302	0.962	(0.906-1.023)	0.272
	MCP3	6	1.144	(1.024-1.279)	0.017	1.008	(1.009,1.043)	0.002	1.230	(0.676-2.236)	0.535	1.127	(0.987-1.288)	0.078	1.122	(0.947-1.329)	0.240	1.125	(0.971-1.304)	0.177
	MIG	6	1.016	(0.956-1.080)	0.607	1.031	(0.956,1.080)	0.607	1.029	(0.742-1.426)	0.873	1.016	(0.946-1.092)	0.658	1.021	(0.9234-1.128)	0.702	1.032	(0.939-1.134)	0.541
	MIP1a	6	0.967	(0.909-1.030)	0.299	1.033	(0.908,1.029)	0.299	0.737	(0.527-1.029)	0.148	0.965	(0.893-1.042)	0.358	0.967	(0.862-1.086)	0.598	0.962	(0.8701-1.064)	0.488
	MIP1b	6	1.113	(1.068-1.159)	0.000	1.021	(1.068,1.160)	0.000	1.094	(0.874-1.369)	0.478	1.122	(1.067-1.181)	0.000	1.124	(1.050-1.202)	0.020	1.123	(1.055-1.195)	0.015
	RANTES	6	0.963	(0.904-1.026)	0.241	1.033	(0.904,1.025)	0.241	0.822	(0.586-1.152)	0.319	0.973	(0.903-1.049)	0.480	0.975	(0.878-1.082)	0.651	0.977	(0.882-1.081)	0.667
	SDF1a	6	1.053	(1.001-1.099)	0.017	1.022	(1.009,1.099)	0.017	0.923	(0.732-1.164)	0.536	1.046	(0.995-1.004)	0.080	1.046	(0.977-1.121)	0.252	1.044	(0.980-1.113)	0.240
**Growthfactors**																				
	bNGF	5	1.066	(0.994-1.144)	0.074	1.037	(0.994,1.143)	0.074	1.048	(0.744-1.476)	0.807	1.056	(0.978-1.140)	0.168	1.056	(0.951-1.173)	0.365	1.056	(0.953-1.170)	0.360
	FGFBasic	5	1.051	(0.992-1.114)	0.090	1.029	(0.992,1.114)	0.090	0.835	(0.658-1.060)	0.236	1.043	(0.981-1.110)	0.180	1.056	(0.958-1.164)	0.334	1.030	(0.953-1.113)	0.499
	GCSF	5	0.998	(0.952-1.046)	0.942	1.024	(0.952,1.046)	0.942	0.852	(0.675-1.076)	0.271	0.995	(0.941-1.052)	0.857	1.002	(0.931-1.077)	0.969	0.998	(0.934-1.068)	0.966
	HGF	5	1.052	(1.005-1.102)	0.031	1.023	(1.005,1.102)	0.031	1.023	(0.814-1.286)	0.856	1.048	(0.992-1.108)	0.094	1.047	(0.972-1.128)	0.291	1.044	(0.977-1.114)	0.271
	MCSF	5	1.033	(0.949-1.125)	0.450	1.044	(0.949,1.125)	0.450	0.890	(0.587-1.348)	0.620	1.032	(0.934-1.140)	0.542	1.043	(0.907-1.199)	0.588	1.041	(0.922-1.175)	0.555
	PDGFbb	5	0.990	(0.946-1.037)	0.683	1.023	(0.946,1.037)	0.683	0.903	(0.719-1.134)	0.446	0.991	(0.939-1.047)	0.754	0.998	(0.928-1.074)	0.965	0.997	(0.928-1.071)	0.936
	SCF	5	1.048	(1.000-1.097)	0.048	1.023	(1.000,1.096)	0.048	1.093	(0.870-1.373)	0.501	1.051	(0.995-1.109)	0.075	1.053	(0.984-1.128)	0.210	1.053	(0.987-1.124)	0.193
	SCGFb	5	1.005	(0.939-1.076)	0.879	1.036	(0.939,1.077)	0.879	0.808	(0.579-1.127)	0.298	0.999	(0.921-1.083)	0.979	1.011	(0.900-1.136)	0.863	0.989	(0.899-1.089)	0.839
	VEGF	5	0.993	(0.945-1.043)	0.770	1.025	(0.945,1.043)	0.770	0.837	(0.655-1.069)	0.249	0.974	(0.918-1.034)	0.394	0.976	(0.903-1.054)	0.572	0.973	(0.908-1.043)	0.485
**Interleukins**																				
	IL1b	5	0.973	(0.921-1.029)	0.340	1.028	(0.921,1.028)	0.340	0.807	(0.617-1.055)	0.215	0.970	(0.909-1.036)	0.366	0.967	(0.880-1.061)	0.515	0.956	(0.880-1.039)	0.353

IL1RA	5	0.934	(0.872-1.000)	0.051	1.036	(0.871,1.000)	0.051	0.867	(0.620-1.213)	0.466	0.943	(0.868-1.024)	0.163	0.945	(0.846-1.055)	0.372	0.945	(0.852-1.049)	0.348
IL2	5	0.986	(0.919-1.058)	0.699	1.037	(0.919,1.059)	0.699	0.888	(0.631-1.251)	0.546	1.000	(0.921-1.086)	0.999	1.010	(0.893-1.142)	0.883	1.009	(0.902-1.129)	0.884
IL2ra	5	0.905	(0.845-0.969)	0.004	1.036	(0.845,0.969)	0.004	0.763	(0.547-1.064)	0.209	0.900	(0.830-0.977)	0.012	0.876	(0.784-0.978)	0.078	0.893	(0.810-0.984)	0.084
IL4	5	1.035	(0.988-1.084)	0.148	1.024	(0.988,1.084)	0.148	0.894	(0.709-1.126)	0.412	1.043	(0.988-1.101)	0.130	1.066	(0.980-1.159)	0.212	1.042	(0.969-1.121)	0.328
IL5	5	0.875	(0.815-0.940)	0.000	1.037	(0.815,0.940)	0.000	0.865	(0.611-1.226)	0.476	0.881	(0.807-0.962)	0.005	0.887	(0.791-0.995)	0.110	0.886	(0.794-0.988)	0.096
IL6	5	1.032	(0.985-1.081)	0.184	1.024	(0.985,1.081)	0.184	0.864	(0.686-1.087)	0.300	1.039	(0.983-1.097)	0.179	1.050	(0.962-1.146)	0.336	1.027	(0.954-1.106)	0.520
IL7	5	0.877	(0.817-0.941)	0.000	1.037	(0.817,0.942)	0.000	0.744	(0.527-1.052)	0.193	0.879	(0.808-0.956)	0.002	0.882	(0.792-0.983)	0.086	0.880	(0.792-0.979)	0.079
IL8	5	0.965	(0.900-1.035)	0.318	1.037	(0.899,1.035)	0.318	0.841	(0.599-1.181)	0.391	0.961	(0.884-1.045)	0.350	0.966	(0.865-1.079)	0.575	0.953	(0.860-1.056)	0.411
IL9	5	1.007	(0.940-1.079)	0.838	1.036	(0.940,1.079)	0.838	0.843	(0.603-1.180)	0.393	0.995	(0.915-1.081)	0.898	1.001	(0.897-1.117)	0.986	0.992	(0.903-1.089)	0.868
IL10	5	1.027	(0.966-1.092)	0.392	1.030	#VALUE!	0.392	0.780	(0.616-0.988)	0.131	1.005	(0.948-1.065)	0.871	1.000	(0.932-1.073)	0.994	0.998	(0.941-1.059)	0.958
IL12	5	1.054	(1.006-1.104)	0.026	1.024	(1.006,1.104)	0.026	0.843	(0.671-1.059)	0.238	1.043	(0.988-1.101)	0.130	1.044	(0.972-1.121)	0.303	1.035	(0.971-1.103)	0.349
IL13	5	0.977	(0.911-1.048)	0.512	1.037	(0.911,1.047)	0.512	0.815	(0.580-1.145)	0.324	0.961	(0.884-1.044)	0.345	0.960	(0.867-1.064)	0.481	0.960	(0.873-1.056)	0.446
IL16	5	1.058	(0.986-1.135)	0.115	1.037	(0.986,1.135)	0.115	1.121	(0.795-1.581)	0.562	1.061	(0.979-1.151)	0.149	1.063	(0.952-1.187)	0.337	1.070	(0.960-1.192)	0.290
IL17	5	1.038	(0.989-1.088)	0.129	1.024	(0.989,1.089)	0.129	0.964	(0.761-1.220)	0.779	1.045	(0.985-1.108)	0.142	1.051	(0.973-1.137)	0.276	1.051	(0.975-1.134)	0.264
IL18	5	1.019	(0.951-1.092)	0.584	1.036	(0.951,1.092)	0.584	0.989	(0.707-1.383)	0.952	1.021	(0.940-1.108)	0.627	1.018	(0.916-1.132)	0.755	1.022	(0.933-1.119)	0.667
**others**																			
IFNg	5	1.028	(0.980-1.078)	0.257	1.024	(0.980,1.078)	0.257	0.947	(0.748-1.200)	0.684	1.024	(0.971-1.081)	0.383	1.047	(0.970-1.130)	0.306	1.015	(0.946-1.090)	0.693
MIF	5	0.978	(0.911-1.050)	0.536	1.037	(0.911,1.049)	0.536	0.877	(0.622-1.238)	0.510	1.001	(0.922-1.086)	0.983	1.003	(0.893-1.127)	0.960	1.002	(0.895-1.123)	0.969
TNFa	5	0.978	(0.911-1.050)	0.536	1.037	(0.911,1.049)	0.536	0.877	(0.622-1.238)	0.510	1.001	(0.923-1.085)	0.983	1.003	(0.895-1.124)	1.124	1.002	(0.890-1.129)	0.970
TNFb	5	0.826	(0.744-0.971)	0.000	1.054	(0.745,0.918)	0.000	0.630	(0.377-1.052)	0.176	0.815	(0.721-0.920)	0.001	0.819	(0.698-0.962)	0.072	0.812	(0.704-0.938)	0.047
TRAIL	5	0.998	(0.953-1.046)	0.941	1.024	(0.953,1.046)	0.941	0.966	(0.768-1.215)	0.784	1.005	(0.950-1.062)	0.871	1.006	(0.935-1.083)	0.875	1.007	(0.940-1.079)	0.851

Abbreviations: CI, Confidence interval; pval, p-value; SNPs, single nucleotide polymorphisms

OR and 95% CI represent change in SD of Inflammatory regulators per log odds increase in cirrhosis.

After correcting for multiple comparison, p-value < 0.05/41 = 0.0012 was considered as significant.

Supplementary Table S5. Pleiotropy and heterogeneity tests of cirrhosis on forty-one inflammatory cytokines for Dataset 1.

Category	Exposure	Pleiotropy Test				Heterogeneity Test		Recommended Method
		MR Egger			PRESSO	Q1	Q2	
		Intercept	SE	Pval	Pval	Pval	Pval	
**Chemokines**	CTACK	-0.002	0.062	0.977	0.186	1.000	0.998	IVW
	Eotaxin	0.068	0.044	0.195	0.718	0.651	0.925	IVW
	GROa	0.085	0.064	0.251	0.862	0.815	0.979	IVW
	IP10	-0.017	0.062	0.796	0.998	0.998	0.995	IVW
	MCP1	0.016	0.043	0.725	0.999	0.999	0.999	IVW
	MCP3	-0.027	0.114	0.822	0.891	0.848	0.744	IVW
	MIG	-0.005	0.062	0.943	0.990	0.995	0.983	IVW
	MIP1α	0.103	0.063	0.180	0.673	0.635	0.941	IVW
	MIP1β	0.007	0.043	0.886	0.988	0.989	0.967	IVW
	RANTES	0.060	0.064	0.403	0.933	0.941	0.985	IVW
	SDF1α	0.050	0.044	0.320	0.951	0.921	0.998	IVW
**Growthfactors**	bNGF	0.007	0.064	0.925	0.988	0.987	0.955	IVW
	FGFBasic	0.087	0.045	0.149	0.278	0.222	0.573	IVW
	GCSF	0.060	0.044	0.267	0.754	0.759	0.999	IVW
	HGF	0.010	0.043	0.824	0.985	0.987	0.964	IVW
	MCSF	0.056	0.078	0.523	0.716	0.682	0.620	IVW
	PDGFbb	0.035	0.043	0.478	0.846	0.892	0.928	IVW
	SCF	-0.016	0.043	0.735	0.987	0.984	0.971	IVW
	SCGFβ	0.082	0.062	0.280	0.785	0.765	0.991	IVW
	VEGF	0.064	0.046	0.258	0.666	0.622	0.877	IVW
**Interleukins**	IL1β	0.071	0.050	0.256	0.555	0.534	0.758	IVW
	IL1RA	0.028	0.063	0.688	0.976	0.986	0.985	IVW
	IL2	0.039	0.064	0.584	0.918	0.941	0.939	IVW
	IL2RA	0.064	0.062	0.379	0.564	0.488	0.497	IVW
	IL4	0.055	0.044	0.294	0.683	0.712	0.915	IVW
	IL5	0.004	0.065	0.952	0.996	0.996	0.982	IVW
	IL6	0.067	0.043	0.219	0.484	0.457	0.743	IVW
	IL7	0.061	0.065	0.413	0.921	0.921	0.999	IVW
	IL8	0.052	0.064	0.477	0.856	0.842	0.861	IVW
	IL9	0.067	0.063	0.367	0.891	0.885	0.999	IVW
	IL10	0.104	0.045	0.102	0.252	0.162	0.775	IVW
	IL12	0.084	0.043	0.145	0.494	0.399	0.977	IVW
	IL13	0.068	0.064	0.365	0.895	0.864	0.985	IVW
	IL16	-0.022	0.065	0.760	0.955	0.942	0.882	IVW
	IL17	0.028	0.045	0.575	0.835	0.872	0.840	IVW
	IL18	0.011	0.063	0.867	0.981	0.970	0.919	IVW
**others**	IFNγ	0.031	0.045	0.539	0.961	0.989	0.962	IVW
	MIF	0.041	0.065	0.573	0.873	0.909	0.895	IVW
	TNFα	0.041	0.065	0.573	0.872	0.909	0.895	IVW
	TNFβ	0.102	0.097	0.367	0.858	0.796	0.907	IVW
	TRAIL	0.013	0.043	0.791	0.980	0.989	0.973	IVW

Q1 pval: p value of Q test from IVW method; Q2 pval: p value of Q test from MR-Egger method
Abbreviations: pval, p-value; Q, Cochran Q statistics; SNPs, single nucleotide polymorphisms; IVW, the inverse variance weighted method.

Supplementary Table S6. Details of cirrhosis predicting SNPs with inflammatory cytokines for Dataset 1.

outcome	SNP	effect allele	other allele	Log (OR)	EAF	se	pval	F
**CTACK**	rs1010022	G	A	0.3943	0.2296	0.0402	1.05E-22	96.2
	rs12484530	A	G	0.3323	0.1124	0.0535	5.13E-10	38.6
	rs56373884	A	G	0.3637	0.2344	0.0397	5.37E-20	83.9
	rs738408	T	C	0.46	0.2268	0.0407	1.47E-29	127.7
	rs8142145	C	T	0.3943	0.2296	0.0402	1.05E-22	96.2
	rs9626087	T	C	0.2401	0.2568	0.038	2.67E-10	39.9
**Eotaxin**	rs1010022	G	A	0.3943	0.2296	0.0402	1.05E-22	96.2
	rs12484530	A	G	0.3323	0.1124	0.0535	5.13E-10	38.6
	rs56373884	A	G	0.3637	0.2344	0.0397	5.37E-20	83.9
	rs738408	T	C	0.46	0.2268	0.0407	1.47E-29	127.7
	rs8142145	C	T	0.3943	0.2296	0.0402	1.05E-22	96.2
	rs9626087	T	C	0.2401	0.2568	0.038	2.67E-10	39.9
**GROa**	rs1010022	G	A	0.3943	0.2296	0.0402	1.05E-22	96.2
	rs12484530	A	G	0.3323	0.1124	0.0535	5.13E-10	38.6
	rs56373884	A	G	0.3637	0.2344	0.0397	5.37E-20	83.9
	rs738408	T	C	0.46	0.2268	0.0407	1.47E-29	127.7
	rs8142145	C	T	0.3943	0.2296	0.0402	1.05E-22	96.2
	rs9626087	T	C	0.2401	0.2568	0.038	2.67E-10	39.9
**IP10**	rs1010022	G	A	0.3943	0.2296	0.0402	1.05E-22	96.2
	rs12484530	A	G	0.3323	0.1124	0.0535	5.13E-10	38.6
	rs56373884	A	G	0.3637	0.2344	0.0397	5.37E-20	83.9
	rs738408	T	C	0.46	0.2268	0.0407	1.47E-29	127.7
	rs8142145	C	T	0.3943	0.2296	0.0402	1.05E-22	96.2
	rs9626087	T	C	0.2401	0.2568	0.038	2.67E-10	39.9
**MCP1**	rs1010022	G	A	0.3943	0.2296	0.0402	1.05E-22	96.2
	rs12484530	A	G	0.3323	0.1124	0.0535	5.13E-10	38.6
	rs56373884	A	G	0.3637	0.2344	0.0397	5.37E-20	83.9
	rs738408	T	C	0.46	0.2268	0.0407	1.47E-29	127.7
	rs8142145	C	T	0.3943	0.2296	0.0402	1.05E-22	96.2
	rs9626087	T	C	0.2401	0.2568	0.038	2.67E-10	39.9
**MCP3**	rs1010022	G	A	0.3943	0.2296	0.0402	1.05E-22	96.0
	rs12484530	A	G	0.3323	0.1124	0.0535	5.13E-10	38.5
	rs56373884	A	G	0.3637	0.2344	0.0397	5.37E-20	83.7
	rs738408	T	C	0.46	0.2268	0.0407	1.47E-29	127.4
	rs8142145	C	T	0.3943	0.2296	0.0402	1.05E-22	96.0
	rs9626087	T	C	0.2401	0.2568	0.038	2.67E-10	39.8
**MIG**	rs1010022	G	A	0.3943	0.1978	0.0402	1.05E-22	96.2
	rs12484530	A	G	0.3323	0.0875	0.0535	5.13E-10	38.6
	rs56373884	A	G	0.3637	0.1869	0.0397	5.37E-20	83.9
	rs738408	T	C	0.46	0.2256	0.0407	1.47E-29	127.7
	rs8142145	C	T	0.3943	0.1978	0.0402	1.05E-22	96.2
	rs9626087	T	C	0.2401	0.2326	0.038	2.67E-10	39.9
**MIP1α**	rs1010022	G	A	0.3943	0.2296	0.0402	1.05E-22	96.2
	rs12484530	A	G	0.3323	0.1124	0.0535	5.13E-10	38.6
	rs56373884	A	G	0.3637	0.2344	0.0397	5.37E-20	83.9
	rs738408	T	C	0.46	0.2268	0.0407	1.47E-29	127.7
	rs8142145	C	T	0.3943	0.2296	0.0402	1.05E-22	96.2
	rs9626087	T	C	0.2401	0.2568	0.038	2.67E-10	39.9
**MIP1β**	rs1010022	G	A	0.3943	0.2296	0.0402	1.05E-22	96.2
	rs12484530	A	G	0.3323	0.1124	0.0535	5.13E-10	38.6

	rs56373884	A	G	0.3637	0.2344	0.0397	5.37E-20	83.9
	rs738408	T	C	0.46	0.2268	0.0407	1.47E-29	127.7
	rs8142145	C	T	0.3943	0.2296	0.0402	1.05E-22	96.2
	rs9626087	T	C	0.2401	0.2568	0.038	2.67E-10	39.9
**RANTES**	rs1010022	G	A	0.3943	0.2296	0.0402	1.05E-22	96.1
	rs12484530	A	G	0.3323	0.1124	0.0535	5.13E-10	38.6
	rs56373884	A	G	0.3637	0.2344	0.0397	5.37E-20	83.9
	rs738408	T	C	0.46	0.2268	0.0407	1.47E-29	127.7
	rs8142145	C	T	0.3943	0.2296	0.0402	1.05E-22	96.1
	rs9626087	T	C	0.2401	0.2568	0.038	2.67E-10	39.9
**SDF1α**	rs1010022	G	A	0.3943	0.2296	0.0402	1.05E-22	96.2
	rs12484530	A	G	0.3323	0.1124	0.0535	5.13E-10	38.6
	rs56373884	A	G	0.3637	0.2344	0.0397	5.37E-20	83.9
	rs738408	T	C	0.46	0.2268	0.0407	1.47E-29	127.7
	rs8142145	C	T	0.3943	0.2296	0.0402	1.05E-22	96.2
	rs9626087	T	C	0.2401	0.2568	0.038	2.67E-10	39.9
**bNGF**	rs12484530	A	G	0.3323	0.1124	0.0535	5.13E-10	38.6
	rs56373884	A	G	0.3637	0.2344	0.0397	5.37E-20	83.9
	rs738408	T	C	0.46	0.2268	0.0407	1.47E-29	127.7
	rs8142145	C	T	0.3943	0.2296	0.0402	1.05E-22	96.2
	rs9626087	T	C	0.2401	0.2568	0.038	2.67E-10	39.9
**FGFBasic**	rs12484530	A	G	0.3323	0.1124	0.0535	5.13E-10	38.6
	rs56373884	A	G	0.3637	0.2344	0.0397	5.37E-20	83.9
	rs738408	T	C	0.46	0.2268	0.0407	1.47E-29	127.7
	rs8142145	C	T	0.3943	0.2296	0.0402	1.05E-22	96.2
	rs9626087	T	C	0.2401	0.2568	0.038	2.67E-10	39.9
**GCSF**	rs12484530	A	G	0.3323	0.1124	0.0535	5.13E-10	38.6
	rs56373884	A	G	0.3637	0.2344	0.0397	5.37E-20	83.9
	rs738408	T	C	0.46	0.2268	0.0407	1.47E-29	127.7
	rs8142145	C	T	0.3943	0.2296	0.0402	1.05E-22	96.2
	rs9626087	T	C	0.2401	0.2568	0.038	2.67E-10	39.9
**HGF**	rs12484530	A	G	0.3323	0.1124	0.0535	5.13E-10	38.6
	rs56373884	A	G	0.3637	0.2344	0.0397	5.37E-20	83.9
	rs738408	T	C	0.46	0.2268	0.0407	1.47E-29	127.7
	rs8142145	C	T	0.3943	0.2296	0.0402	1.05E-22	96.2
	rs9626087	T	C	0.2401	0.2568	0.038	2.67E-10	39.9
**MCSF**	rs12484530	A	G	0.3323	0.1124	0.0535	5.13E-10	38.5
	rs56373884	A	G	0.3637	0.2344	0.0397	5.37E-20	83.7
	rs738408	T	C	0.46	0.2268	0.0407	1.47E-29	127.4
	rs8142145	C	T	0.3943	0.2296	0.0402	1.05E-22	96.0
	rs9626087	T	C	0.2401	0.2568	0.038	2.67E-10	39.8
**PDGFbb**	rs12484530	A	G	0.3323	0.1124	0.0535	5.13E-10	38.6
	rs56373884	A	G	0.3637	0.2344	0.0397	5.37E-20	83.9
	rs738408	T	C	0.46	0.2268	0.0407	1.47E-29	127.7
	rs8142145	C	T	0.3943	0.2296	0.0402	1.05E-22	96.2
	rs9626087	T	C	0.2401	0.2568	0.038	2.67E-10	39.9
**SCF**	rs12484530	A	G	0.3323	0.1124	0.0535	5.13E-10	38.6
	rs56373884	A	G	0.3637	0.2344	0.0397	5.37E-20	83.9
	rs738408	T	C	0.46	0.2268	0.0407	1.47E-29	127.7
	rs8142145	C	T	0.3943	0.2296	0.0402	1.05E-22	96.2
	rs9626087	T	C	0.2401	0.2568	0.038	2.67E-10	39.9
**SCGFβ**	rs12484530	A	G	0.3323	0.1124	0.0535	5.13E-10	38.6
	rs56373884	A	G	0.3637	0.2344	0.0397	5.37E-20	83.9
	rs738408	T	C	0.46	0.2268	0.0407	1.47E-29	127.7

	rs8142145	C	T	0.3943	0.2296	0.0402	1.05E-22	96.2
	rs9626087	T	C	0.2401	0.2568	0.038	2.67E-10	39.9
**VEGF**	rs12484530	A	G	0.3323	0.1124	0.0535	5.13E-10	38.6
	rs56373884	A	G	0.3637	0.2344	0.0397	5.37E-20	83.9
	rs738408	T	C	0.46	0.2268	0.0407	1.47E-29	127.7
	rs8142145	C	T	0.3943	0.2296	0.0402	1.05E-22	96.2
	rs9626087	T	C	0.2401	0.2568	0.038	2.67E-10	39.9
**IL1b**	rs12484530	A	G	0.3323	0.1124	0.0535	5.13E-10	38.6
	rs56373884	A	G	0.3637	0.2344	0.0397	5.37E-20	83.9
	rs738408	T	C	0.46	0.2268	0.0407	1.47E-29	127.7
	rs8142145	C	T	0.3943	0.2296	0.0402	1.05E-22	96.1
	rs9626087	T	C	0.2401	0.2568	0.038	2.67E-10	39.9
**IL1RA**	rs12484530	A	G	0.3323	0.1124	0.0535	5.13E-10	38.6
	rs56373884	A	G	0.3637	0.2344	0.0397	5.37E-20	83.9
	rs738408	T	C	0.46	0.2268	0.0407	1.47E-29	127.7
	rs8142145	C	T	0.3943	0.2296	0.0402	1.05E-22	96.2
	rs9626087	T	C	0.2401	0.2568	0.038	2.67E-10	39.9
**IL2**	rs12484530	A	G	0.3323	0.1124	0.0535	5.13E-10	38.6
	rs56373884	A	G	0.3637	0.2344	0.0397	5.37E-20	83.9
	rs738408	T	C	0.46	0.2268	0.0407	1.47E-29	127.7
	rs8142145	C	T	0.3943	0.2296	0.0402	1.05E-22	96.2
	rs9626087	T	C	0.2401	0.2568	0.038	2.67E-10	39.9
**IL2RA**	rs12484530	A	G	0.3323	0.1124	0.0535	5.13E-10	38.6
	rs56373884	A	G	0.3637	0.2344	0.0397	5.37E-20	83.9
	rs738408	T	C	0.46	0.2268	0.0407	1.47E-29	127.7
	rs8142145	C	T	0.3943	0.2296	0.0402	1.05E-22	96.2
	rs9626087	T	C	0.2401	0.2568	0.038	2.67E-10	39.9
**IL4**	rs12484530	A	G	0.3323	0.1124	0.0535	5.13E-10	38.6
	rs56373884	A	G	0.3637	0.2344	0.0397	5.37E-20	83.9
	rs738408	T	C	0.46	0.2268	0.0407	1.47E-29	127.7
	rs8142145	C	T	0.3943	0.2296	0.0402	1.05E-22	96.2
	rs9626087	T	C	0.2401	0.2568	0.038	2.67E-10	39.9
**IL5**	rs12484530	A	G	0.3323	0.1124	0.0535	5.13E-10	38.6
	rs56373884	A	G	0.3637	0.2344	0.0397	5.37E-20	83.9
	rs738408	T	C	0.46	0.2268	0.0407	1.47E-29	127.7
	rs8142145	C	T	0.3943	0.2296	0.0402	1.05E-22	96.1
	rs9626087	T	C	0.2401	0.2568	0.038	2.67E-10	39.9
**IL6**	rs12484530	A	G	0.3323	0.1124	0.0535	5.13E-10	38.6
	rs56373884	A	G	0.3637	0.2344	0.0397	5.37E-20	83.9
	rs738408	T	C	0.46	0.2268	0.0407	1.47E-29	127.7
	rs8142145	C	T	0.3943	0.2296	0.0402	1.05E-22	96.2
	rs9626087	T	C	0.2401	0.2568	0.038	2.67E-10	39.9
**IL7**	rs12484530	A	G	0.3323	0.1124	0.0535	5.13E-10	38.6
	rs56373884	A	G	0.3637	0.2344	0.0397	5.37E-20	83.9
	rs738408	T	C	0.46	0.2268	0.0407	1.47E-29	127.7
	rs8142145	C	T	0.3943	0.2296	0.0402	1.05E-22	96.1
	rs9626087	T	C	0.2401	0.2568	0.038	2.67E-10	39.9
**IL8**	rs12484530	A	G	0.3323	0.1124	0.0535	5.13E-10	38.6
	rs56373884	A	G	0.3637	0.2344	0.0397	5.37E-20	83.9
	rs738408	T	C	0.46	0.2268	0.0407	1.47E-29	127.7
	rs8142145	C	T	0.3943	0.2296	0.0402	1.05E-22	96.2
	rs9626087	T	C	0.2401	0.2568	0.038	2.67E-10	39.9
**IL9**	rs12484530	A	G	0.3323	0.1124	0.0535	5.13E-10	38.6
	rs56373884	A	G	0.3637	0.2344	0.0397	5.37E-20	83.9

	rs738408	T	C	0.46	0.2268	0.0407	1.47E-29	127.7
	rs8142145	C	T	0.3943	0.2296	0.0402	1.05E-22	96.2
	rs9626087	T	C	0.2401	0.2568	0.038	2.67E-10	39.9
**IL10**	rs12484530	A	G	0.3323	0.1124	0.0535	5.13E-10	38.6
	rs56373884	A	G	0.3637	0.2344	0.0397	5.37E-20	83.9
	rs738408	T	C	0.46	0.2268	0.0407	1.47E-29	127.7
	rs8142145	C	T	0.3943	0.2296	0.0402	1.05E-22	96.2
	rs9626087	T	C	0.2401	0.2568	0.038	2.67E-10	39.9
**IL12**	rs12484530	A	G	0.3323	0.1124	0.0535	5.13E-10	38.6
	rs56373884	A	G	0.3637	0.2344	0.0397	5.37E-20	83.9
	rs738408	T	C	0.46	0.2268	0.0407	1.47E-29	127.7
	rs8142145	C	T	0.3943	0.2296	0.0402	1.05E-22	96.2
	rs9626087	T	C	0.2401	0.2568	0.038	2.67E-10	39.9
**IL13**	rs12484530	A	G	0.3323	0.1124	0.0535	5.13E-10	38.6
	rs56373884	A	G	0.3637	0.2344	0.0397	5.37E-20	83.9
	rs738408	T	C	0.46	0.2268	0.0407	1.47E-29	127.7
	rs8142145	C	T	0.3943	0.2296	0.0402	1.05E-22	96.2
	rs9626087	T	C	0.2401	0.2568	0.038	2.67E-10	39.9
**IL16**	rs12484530	A	G	0.3323	0.1124	0.0535	5.13E-10	38.6
	rs56373884	A	G	0.3637	0.2344	0.0397	5.37E-20	83.9
	rs738408	T	C	0.46	0.2268	0.0407	1.47E-29	127.7
	rs8142145	C	T	0.3943	0.2296	0.0402	1.05E-22	96.2
	rs9626087	T	C	0.2401	0.2568	0.038	2.67E-10	39.9
**IL17**	rs12484530	A	G	0.3323	0.1124	0.0535	5.13E-10	38.6
	rs56373884	A	G	0.3637	0.2344	0.0397	5.37E-20	83.9
	rs738408	T	C	0.46	0.2268	0.0407	1.47E-29	127.7
	rs8142145	C	T	0.3943	0.2296	0.0402	1.05E-22	96.2
	rs9626087	T	C	0.2401	0.2568	0.038	2.67E-10	39.9
**IL18**	rs12484530	A	G	0.3323	0.1124	0.0535	5.13E-10	38.6
	rs56373884	A	G	0.3637	0.2344	0.0397	5.37E-20	83.9
	rs738408	T	C	0.46	0.2268	0.0407	1.47E-29	127.7
	rs8142145	C	T	0.3943	0.2296	0.0402	1.05E-22	96.2
	rs9626087	T	C	0.2401	0.2568	0.038	2.67E-10	39.9
**IFNγ**	rs12484530	A	G	0.3323	0.1124	0.0535	5.13E-10	38.6
	rs56373884	A	G	0.3637	0.2344	0.0397	5.37E-20	83.9
	rs738408	T	C	0.46	0.2268	0.0407	1.47E-29	127.7
	rs8142145	C	T	0.3943	0.2296	0.0402	1.05E-22	96.2
	rs9626087	T	C	0.2401	0.2568	0.038	2.67E-10	39.9
**MIF**	rs12484530	A	G	0.3323	0.1124	0.0535	5.13E-10	38.6
	rs56373884	A	G	0.3637	0.2344	0.0397	5.37E-20	83.9
	rs738408	T	C	0.46	0.2268	0.0407	1.47E-29	127.7
	rs8142145	C	T	0.3943	0.2296	0.0402	1.05E-22	96.2
	rs9626087	T	C	0.2401	0.2568	0.038	2.67E-10	39.9
**TNFα**	rs12484530	A	G	0.3323	0.1124	0.0535	5.13E-10	38.6
	rs56373884	A	G	0.3637	0.2344	0.0397	5.37E-20	83.9
	rs738408	T	C	0.46	0.2268	0.0407	1.47E-29	127.7
	rs8142145	C	T	0.3943	0.2296	0.0402	1.05E-22	96.2
	rs9626087	T	C	0.2401	0.2568	0.038	2.67E-10	39.9
**TNFβ**	rs12484530	A	G	0.3323	0.1124	0.0535	5.13E-10	38.5
	rs56373884	A	G	0.3637	0.2344	0.0397	5.37E-20	83.8
	rs738408	T	C	0.46	0.2268	0.0407	1.47E-29	127.6
	rs8142145	C	T	0.3943	0.2296	0.0402	1.05E-22	96.1
	rs9626087	T	C	0.2401	0.2568	0.038	2.67E-10	39.9
**TRAIL**	rs12484530	A	G	0.3323	0.1124	0.0535	5.13E-10	38.6

rs56373884	A	G	0.3637	0.2344	0.0397	5.37E-20	83.9
rs738408	T	C	0.46	0.2268	0.0407	1.47E-29	127.7
rs8142145	C	T	0.3943	0.2296	0.0402	1.05E-22	96.2
rs9626087	T	C	0.2401	0.2568	0.038	2.67E-10	39.9

Abbreviations: EAF: effect allele frequency, Log (OR): log odds ratio, se: standard error;
Log (OR) for cirrhosis represents log (OR) change in cirrhosis risk per 1 copy of effect allele.

Supplementary Table S7. MR estimates for Dataset 2.

Category	Exposure	No. of SNPs	IVW			MR-Lasso			MR-Egger			Weighted Median			Simple Mode			Weighted Mode		
			OR	95% CI	pval	OR	95% CI	pval	OR	95% CI	pval	OR	95% CI	pval	OR	95% CI	pval	OR	95% CI	pval
**Chemokines**																				
	CTACK	7	1.032	(0.992,1.074)	0.122	1.020	(0.993,1.075)	0.111	0.693	(0.270,1.782)	0.481	1.032	(0.986,1.079)	0.174	0.803	(0.607,1.061)	0.174	1.035	(0.993,1.078)	0.156
	Eotaxin	11	0.994	(0.971,1.018)	0.641	1.012	(0.971,1.018)	0.641	1.453	(1.009,2.092)	0.075	0.995	(0.963,1.028)	0.758	0.792	(0.651,0.965)	0.043	0.995	(0.967,1.024)	0.757
	GROa	6	0.993	(0.984,1.003)	0.162	1.037	(0.970,1.120)	0.254	0.953	(0.920,0.987)	0.056	0.993	(0.984,1.003)	0.172	1.054	(0.980,1.133)	0.214	0.994	(0.984,1.003)	0.247
	IP10	3	0.741	(0.684,0.802)	0.000	1.042	(0.687,0.805)	0.000	0.752	(0.609,0.928)	0.229	0.741	(0.626,0.877)	0.000	0.741	(0.484,1.134)	0.302	0.741	(0.642,0.856)	0.055
	MCP1	19	0.993	(0.986,1.001)	0.092	1.004	(0.986,1.001)	0.092	0.975	(0.957,0.993)	0.028	0.994	(0.984,1.003)	0.184	0.834	(0.700,0.994)	0.073	0.991	(0.968,1.014)	0.469
	MCP3	5	1.021	(0.988,1.054)	0.021	1.001	(1.001,1.006)	0.011	1.078	(0.969,1.199)	0.030	1.020	(0.982,1.060)	0.299	1.164	(1.057,1.281)	0.054	1.022	(0.986,1.060)	0.322
	MIG	13	0.995	(0.988,1.001)	0.123	1.003	(0.989,1.001)	0.090	1.016	(0.996,1.035)	0.140	0.994	(0.986,1.001)	0.101	0.995	(0.967,1.023)	0.716	0.995	(0.985,1.004)	0.276
	MIP1a	7	0.987	(0.967,1.007)	0.204	1.010	(0.968,1.007)	0.189	0.949	(0.901,0.998)	0.099	0.986	(0.961,1.012)	0.291	1.036	(0.908,1.184)	0.616	0.984	(0.964,1.005)	0.189
	MIP1b	15	0.999	(0.987,1.011)	0.839	1.006	(0.988,1.011)	0.920	1.031	(1.005,1.056)	0.033	1.000	(0.986,1.014)	0.968	1.012	(0.941,1.088)	0.755	1.000	(0.988,1.011)	0.955
	RANTES	10	1.009	(1.000,1.018)	0.390	1.004	(0.998,1.015)	0.141	1.062	(0.894,1.260)	0.513	1.009	(0.999,1.020)	0.846	0.812	(0.625,1.055)	0.153	1.009	(0.993,1.025)	0.310
	SDF1a	4	0.912	(0.818,1.016)	0.095	1.057	(0.817,1.015)	0.092	0.825	(0.427,1.593)	0.625	0.909	(0.790,1.047)	0.186	0.927	(0.737,1.164)	0.560	0.907	(0.812,1.013)	0.183
**Growthfactors**																				
	bNGF	7	0.992	(0.981,1.003)	0.133	1.006	(0.980,1.003)	0.133	1.566	(1.009,2.430)	0.102	0.992	(0.979,1.004)	0.190	1.055	(0.974,1.143)	0.238	0.992	(0.979,1.006)	0.299
	FGFBasic	5	0.949	(0.861,1.045)	0.287	1.127	(0.691,1.107)	0.267	0.513	(0.225,1.173)	0.212	0.944	(0.846,1.054)	0.306	1.053	(0.837,1.324)	0.684	0.942	(0.849,1.045)	0.323
	GCSF	8	1.005	(1.000,1.010)	0.379	1.103	(0.841,1.236)	0.842	1.014	(1.004,1.024)	0.315	1.005	(0.999,1.012)	0.893	0.876	(0.701,1.096)	0.284	1.002	(0.990,1.015)	0.738
	HGF	7	1.021	(0.982,1.062)	0.287	1.097	(0.892,1.287)	0.458	0.936	(0.840,1.043)	0.283	1.039	(0.994,1.087)	0.093	0.921	(0.836,1.015)	0.149	1.032	(0.991,1.074)	0.176
	MCSF	8	1.003	(1.001,1.006)	0.011	1.023	(1.002,1.096)	0.042	1.065	(1.009,1.125)	0.062	1.003	(1.000,1.007)	0.044	1.029	(0.995,1.064)	0.140	1.003	(0.994,1.013)	0.508
	PDGFbb	11	1.010	(0.999,1.020)	0.081	1.049	(0.987,1.191)	0.090	0.990	(0.954,1.027)	0.609	1.012	(1.000,1.024)	0.055	1.023	(0.974,1.074)	0.390	1.008	(0.997,1.019)	0.196
	SCF	8	0.945	(0.886,1.009)	0.089	1.094	(0.979,1.392)	0.084	0.942	(0.849,1.046)	0.307	0.938	(0.870,1.011)	0.096	1.061	(0.861,1.306)	0.597	0.941	(0.872,1.016)	0.163
	SCGFb	14	1.013	(0.992,1.034)	0.235	1.010	(0.991,1.034)	0.251	1.070	(1.009,1.135)	0.043	1.014	(0.985,1.043)	0.344	0.900	(0.818,0.992)	0.053	1.017	(0.991,1.044)	0.223
	VEGF	9	0.921	(0.911,0.932)	0.426	1.034	(0.931,1.058)	0.803	1.003	(0.921,1.092)	0.951	0.922	(0.916,0.927)	0.286	0.769	(0.572,1.035)	0.121	0.923	(0.914,0.933)	0.306
**Interleukins**																				
	IL1b	6	1.032	(0.992,1.073)	0.115	1.020	(0.992,1.073)	0.115	1.132	(0.956,1.340)	0.223	1.038	(0.981,1.097)	0.196	0.926	(0.808,1.060)	0.315	1.036	(0.991,1.084)	0.180

	IL1RA	9	1.024	(0.999,1.049)	0.059	1.012	(0.999,1.049)	0.055	1.004	(0.947,1.064)	0.899	1.027	(0.998,1.057)	0.066	0.884	(0.798,0.979)	0.046	1.024	(0.997,1.051)	0.114
	IL2	9	0.984	(0.904,1.072)	0.719	1.043	(0.915,1.080)	0.889	0.970	(0.820,1.147)	0.730	1.009	(0.922,1.105)	0.843	0.884	(0.758,1.032)	0.156	0.998	(0.916,1.087)	0.962
	IL2ra	6	1.046	(0.992,1.103)	0.096	1.047	(0.882,1.058)	0.449	1.088	(0.515,2.296)	0.836	1.047	(0.983,1.116)	0.152	1.175	(0.934,1.477)	0.227	1.047	(0.985,1.113)	0.197
	IL4	9	1.025	(0.776,1.352)	0.864	1.114	(0.890,1.358)	0.376	1.149	(0.738,1.791)	0.558	1.053	(0.749,1.478)	0.768	0.697	(0.176,2.763)	0.621	1.109	(0.372,3.306)	0.857
	IL5	4	1.010	(1.002,1.018)	0.016	1.004	(1.002,1.018)	0.015	0.999	(0.974,1.024)	0.935	1.010	(1.000,1.021)	0.054	1.004	(0.979,1.029)	0.791	1.011	(1.002,1.020)	0.098
	IL6	4	0.945	(0.858,1.041)	0.255	1.034	(0.950,1.079)	0.704	0.701	(0.304,1.619)	0.493	0.945	(0.847,1.056)	0.319	1.294	(0.887,1.888)	0.273	0.934	(0.844,1.033)	0.276
	IL7	10	1.002	(0.988,1.017)	0.752	1.007	(0.988,1.017)	0.752	1.043	(1.013,1.074)	0.229	1.003	(0.988,1.020)	0.674	1.029	(0.975,1.086)	0.322	1.003	(0.990,1.017)	0.658
	IL8	4	1.008	(1.000,1.016)	0.488	1.157	(0.736,1.305)	0.892	0.981	(0.892,1.079)	0.727	1.008	(0.999,1.018)	0.097	1.163	(0.975,1.388)	0.192	1.009	(0.999,1.019)	0.176
	IL9	6	0.980	(0.962,0.999)	0.365	1.081	(0.860,1.166)	0.984	1.000	(0.952,1.051)	0.987	0.976	(0.954,0.998)	0.344	0.971	(0.945,0.999)	0.965	0.977	(0.956,0.998)	0.811
	IL10	11	0.990	(0.953,1.027)	0.589	1.019	(0.954,1.028)	0.609	1.071	(0.990,1.158)	0.120	0.992	(0.944,1.043)	0.762	0.866	(0.737,1.018)	0.112	0.988	(0.950,1.028)	0.571
	IL12	11	0.992	(0.967,1.019)	0.569	1.013	(0.967,1.019)	0.574	1.051	(0.991,1.115)	0.129	0.994	(0.958,1.033)	0.772	0.714	(0.591,0.862)	0.006	0.997	(0.970,1.025)	0.827
	IL13	13	0.994	(0.985,1.003)	0.169	1.005	(0.985,1.003)	0.169	1.131	(1.004,1.274)	0.067	0.995	(0.984,1.006)	0.330	0.855	(0.657,1.113)	0.268	0.996	(0.984,1.009)	0.581
	IL16	10	0.995	(0.968,1.023)	0.740	1.014	(0.971,1.025)	0.883	0.986	(0.920,1.057)	0.696	0.986	(0.950,1.024)	0.475	0.994	(0.948,1.041)	0.793	0.995	(0.963,1.029)	0.791
	IL17	10	0.997	(0.975,1.021)	0.822	1.011	(0.975,1.020)	0.823	1.068	(1.009,1.130)	0.052	0.998	(0.965,1.031)	0.895	0.999	(0.895,1.115)	0.987	0.999	(0.974,1.025)	0.944
	IL18	17	0.987	(0.965,1.010)	0.261	1.011	(0.964,1.007)	0.168	0.994	(0.925,1.068)	0.864	0.979	(0.954,1.005)	0.109	1.084	(0.995,1.182)	0.084	0.982	(0.958,1.007)	0.183
**others**																				
	IFNg	9	0.973	(0.901,1.050)	0.473	1.040	(0.900,1.049)	0.464	1.139	(0.911,1.423)	0.290	0.975	(0.883,1.076)	0.617	0.724	(0.509,1.029)	0.110	0.972	(0.894,1.056)	0.520
	MIF	6	0.976	(0.929,1.026)	0.347	1.079	(0.899,1.210)	0.576	1.068	(0.917,1.244)	0.446	0.975	(0.920,1.034)	0.394	0.953	(0.857,1.059)	0.409	0.967	(0.915,1.022)	0.290
	TNFa	5	1.095	(0.926,1.295)	0.290	1.090	(0.926,1.296)	0.290	0.942	(0.332,2.669)	0.918	1.099	(0.912,1.324)	0.323	1.287	(0.758,2.186)	0.403	1.102	(0.932,1.301)	0.319
	TNFb	6	1.006	(1.001,1.011)	0.114	1.045	(0.967,1.149)	0.235	1.024	(0.981,1.068)	0.343	1.006	(1.000,1.012)	0.514	0.790	(0.674,0.926)	0.333	1.006	(0.998,1.015)	0.212
	TRAIL	26	1.022	(0.974,1.073)	0.366	1.022	(0.964,1.052)	0.761	1.034	(0.926,1.156)	0.565	1.056	(0.990,1.126)	0.096	1.017	(0.903,1.145)	0.787	1.041	(0.961,1.128)	0.350

Abbreviations: CI, Confidence interval; OR, Odds Ratio; pval, p-value; SNPs, single nucleotide polymorphisms

OR and 95% CI represent the change in the odds ratio of cirrhosis per 1 SD increase in inflammatory cytokines.

After correcting for multiple comparison, p-value < 0.05/41 = 0.0012 was considered as significant.

Supplementary Table S8. Pleiotropy and heterogeneity tests for Dataset 2.

Category	Exposure	Pleiotropy Test				Heterogeneity Test		Recommended
		MR_Egger			PRESSO	Q1	Q2	Method
		Intercept	SE	Pval	Pval	Pval	Pval	
**Chemokines**	CTACK	0.159	0.192	0.446	0.995	0.960	0.976	Weighted Median
	Eotaxin	-0.041	0.020	0.071	0.629	0.524	0.842	IVW
	GROa	0.017	0.007	0.077	0.501	0.266	0.931	IVW
	IP10	-0.012	0.077	0.905	0.581	0.981	0.902	IVW
	MCP1	0.007	0.003	0.062	0.517	0.458	0.851	IVW
	MCP3	-0.031	0.029	0.404	0.597	0.694	0.839	IVW
	MIG	-0.003	0.001	0.469	0.346	0.110	0.326	IVW
	MIP1a	0.009	0.005	0.162	0.521	0.376	0.585	IVW
	MIP1b	-0.013	0.005	0.162	0.532	0.309	0.813	IVW
	RANTES	-0.022	0.037	0.578	0.555	0.983	0.978	IVW
	SDF1a	0.008	0.025	0.792	0.967	0.922	0.820	IVW
**Growthfactors**	bNGF	-0.081	0.040	0.097	0.538	0.350	0.771	IVW
	FGFBasic	0.066	0.045	0.239	0.579	0.577	0.865	IVW
	GCSF	-0.005	0.003	0.020	0.511	0.676	0.991	IVW
	HGF	0.009	0.005	0.156	0.226	0.254	0.416	IVW
	MCSF	-0.018	0.008	0.073	0.524	0.502	0.952	IVW
	PDGFbb	0.002	0.002	0.309	0.425	0.237	0.255	IVW
	SCF	0.001	0.009	0.941	0.992	0.950	0.904	IVW
	SCGFb	-0.016	0.008	0.072	0.438	0.510	0.759	IVW
	VEGF	-0.041	0.021	0.090	0.513	0.404	0.121	IVW
**Interleukins**	IL1b	-0.026	0.024	0.331	0.530	0.603	0.660	IVW
	IL1RA	-0.011	0.011	0.404	0.697	0.420	0.398	IVW
	IL2	0.007	0.010	0.496	0.921	0.831	0.806	IVW
	IL2ra	-0.005	0.044	0.923	0.985	0.920	0.839	IVW
	IL4	-0.035	0.054	0.536	0.993	0.980	0.979	IVW
	IL5	0.006	0.006	0.453	0.556	0.446	0.404	IVW
	IL6	0.034	0.049	0.554	0.511	0.696	0.624	IVW
	IL7	-0.009	0.003	0.186	0.436	0.234	0.935	IVW
	IL8	0.017	0.030	0.625	0.643	0.840	0.774	IVW
	IL9	-0.004	0.005	0.423	0.645	0.376	0.349	IVW
	IL10	-0.011	0.005	0.508	0.605	0.694	0.987	IVW
	IL12	-0.010	0.004	0.599	0.694	0.645	0.955	IVW
	IL13	-0.047	0.022	0.553	0.668	0.894	0.999	IVW
	IL16	0.002	0.007	0.771	0.231	0.230	0.171	IVW
	IL17	-0.019	0.007	0.329	0.539	0.378	0.932	IVW
	IL18	-0.001	0.008	0.856	0.887	0.859	0.813	IVW

**others**	IFNg	-0.040	0.027	0.183	0.725	0.844	0.962	IVW
	MIF	-0.014	0.012	0.291	0.785	0.439	0.503	IVW
	TNFa	0.019	0.068	0.793	0.962	0.981	0.952	IVW
	TNFb	-0.003	0.004	0.467	0.579	0.966	0.989	IVW
	TRAIL	-0.001	0.002	0.701	0.354	0.523	0.433	IVW

Q1 pval: p value of Q test from IVW method; Q2 pval: p value of Q test from MR-Egger method Abbreviations: pval, p-value; Q, Cochran Q statistics; SNPs, single nucleotide polymorphisms; IVW, the inverse variance weighted method.

Supplementary Table S9. SNPs information for Dataset 2.

				Inflammatory cytokines			Cirrhosis		
	SNP	effect allele	other allele	beta	se	pval	beta	se	pval
CTACK									
	rs113076930	C	T	-0.3976	0.0832	2.53E-06	-0.0131	0.0082	0.8901
	rs116943377	G	A	-0.2845	0.0613	3.13E-06	-0.0770	0.1212	0.4746
	rs11790474	C	T	-0.1783	0.0312	1.08E-08	0.0502	0.2534	0.1572
	rs12438277	C	T	-0.1542	0.0337	4.91E-06	0.0714	0.4655	0.1219
	rs55764737	C	T	-0.5313	0.0972	4.62E-08	0.0564	0.0889	0.4741
	rs76395525	G	A	-0.5277	0.1083	9.55E-07	0.1393	0.3475	0.3114
	rs79810036	G	A	-0.3791	0.0822	4.87E-06	-0.0816	0.1463	0.4230
Eotaxin									
	rs11920996	C	T	-0.2962	0.0378	5.23E-15	0.1696	2.4970	0.0542
	rs1476670	C	A	0.1007	0.0217	3.51E-06	0.0356	0.0808	0.3409
	rs2040143	G	A	0.0868	0.0178	1.12E-06	-0.0192	0.0239	0.5781
	rs2229593	C	T	-0.3627	0.0407	6.73E-19	0.0834	0.1638	0.3893
	rs2249581	C	T	0.0906	0.018	4.92E-07	0.0169	0.0198	0.6046
	rs2419841	C	T	0.1277	0.0279	4.98E-06	-0.0460	0.0994	0.3567
	rs5754733	C	A	0.1042	0.0214	1.06E-06	-0.0030	0.0016	0.9395
	rs73065695	G	A	-0.1536	0.0282	5.05E-08	0.0377	0.0615	0.4602
	rs75426604	C	A	0.1366	0.0291	2.53E-06	0.0377	0.0722	0.3987
	rs9317045	C	A	-0.1182	0.0237	5.82E-07	-0.0040	0.0024	0.9109
	rs9833459	C	T	0.1125	0.0173	7.68E-11	-0.0315	0.0703	0.3458
GROa									
	rs115214168	C	T	-0.4397	0.0831	1.41E-07	0.0040	0.0019	0.9636
	rs117078153	C	T	0.2605	0.0573	4.06E-06	0.0459	0.0767	0.4507
	rs1361829	G	A	0.1131	0.0243	3.21E-06	0.0090	0.0077	0.7576
	rs2422841	G	A	0.1657	0.0361	4.66E-06	0.0677	0.3570	0.1503
	rs3845622	C	A	0.2407	0.0384	3.51E-10	0.0070	0.0044	0.8887
	rs73020704	G	A	-0.2677	0.0548	1.54E-06	0.0871	0.1634	0.4059
IP10									
	rs143799975	G	A	0.7984	0.1637	1.00E-06	-0.2392	0.0326	0.7707
	rs1951169	G	A	-0.1147	0.0245	2.47E-06	0.0344	0.1039	0.2595
	rs7645625	G	T	0.1086	0.0237	4.41E-06	-0.0492	0.0860	0.1014
MCP1									
	rs112313229	G	A	0.1646	0.0313	1.43E-07	-0.0257	0.0239	0.7176
	rs11920996	C	T	-0.1811	0.0377	1.63E-06	-0.1696	2.4970	0.0542
	rs12062235	G	T	-0.1464	0.0321	4.77E-06	0.1080	0.8650	0.0993
	rs12493953	G	A	0.0935	0.0173	6.98E-08	-0.0129	0.0131	0.6749
	rs12496585	G	T	-0.0963	0.021	4.19E-06	-0.0354	0.0915	0.3009
	rs145775149	G	A	0.5645	0.1239	4.67E-06	-0.2461	4.4384	0.0442
	rs2712431	C	A	0.0787	0.0172	4.76E-06	-0.0377	0.1252	0.2367
	rs77116118	C	T	0.4279	0.0827	2.86E-07	-0.0040	0.0018	0.9761
	rs79939301	G	A	-0.1445	0.0256	1.55E-08	-0.0040	0.0021	0.9485
	rs8020709	G	A	0.0756	0.0164	4.15E-06	-0.0237	0.0408	0.4385
MCP3									
	rs10892381	C	T	0.2412	0.0476	3.56E-07	0.0408	0.1497	0.2150
	rs28394764	T	A	0.6012	0.1282	3.01E-06	0.0139	0.0101	0.8330
	rs6993671	C	T	-0.2065	0.0447	3.77E-06	0.0161	0.0191	0.6006
	rs73669117	G	A	0.6238	0.131	2.56E-06	0.1031	0.1863	0.4202
MIG									

	rs111607343	G	A	0.521	0.1119	2.83E-06	0.0070	0.0038	0.9364
	rs11177248	G	A	-0.3073	0.067	4.45E-06	-0.0516	0.1035	0.3821
	rs112861654	G	A	0.2765	0.0529	1.81E-07	-0.0943	0.9244	0.0813
	rs117831247	C	T	0.8334	0.1754	2.16E-06	-0.0620	0.0655	0.6567
	rs139010077	C	T	-0.4322	0.095	3.55E-06	0.0888	0.1315	0.5006
	rs1796086	C	T	0.2096	0.0403	2.23E-07	0.0583	0.1683	0.2708
	rs41272086	G	A	0.2226	0.0415	7.43E-08	-0.0315	0.0461	0.5054
	rs55876513	G	T	-0.166	0.0255	8.23E-11	-0.0080	0.0060	0.8184
	rs5752128	C	T	0.1685	0.0369	4.34E-06	0.0070	0.0043	0.8953
	rs62562991	G	A	-0.6236	0.126	8.40E-07	0.0346	0.0297	0.7556
	rs6679677	C	A	-0.162	0.0329	8.86E-07	0.0030	0.0015	0.9536
	rs77086208	C	T	-0.3226	0.0698	3.83E-06	0.1960	1.4393	0.1083
	rs816960	C	T	0.1224	0.0244	5.01E-07	-0.0010	0.0004	0.9888
MIP1a									
	rs10835056	G	T	-0.1194	0.0254	2.60E-06	-0.0050	0.0031	0.8933
	rs12690897	G	A	-0.1248	0.0262	2.11E-06	0.0080	0.0061	0.8105
	rs184154340	G	A	-0.331	0.0693	1.86E-06	0.1065	0.5464	0.1545
	rs34771762	G	A	-0.249	0.0523	2.13E-06	-0.0987	0.7422	0.1058
	rs57786342	G	A	-0.1314	0.0285	4.06E-06	-0.0402	0.1141	0.2752
	rs60198979	G	A	0.2146	0.0458	2.62E-06	0.0598	0.1656	0.2818
	rs7232268	G	A	-0.2821	0.0599	2.55E-06	0.0060	0.0032	0.9400
MIP1b									
	rs11130043	G	A	0.0731	0.0157	3.22E-06	0.0121	0.0119	0.6890
	rs113010081	C	T	0.5954	0.0236	3.85E-140	0.0070	0.0044	0.8843
	rs113877493	C	T	0.6124	0.0218	1.62E-173	0.0834	0.4762	0.1390
	rs116237296	G	A	-0.5437	0.1115	7.23E-07	-0.0507	0.0476	0.7126
	rs117453826	G	A	0.5774	0.0593	5.07E-22	0.0592	0.0698	0.6035
	rs141102180	G	T	-0.3225	0.0393	1.08E-16	0.0440	0.0419	0.7055
	rs1437220	C	T	-0.1478	0.0315	3.53E-06	0.0149	0.0110	0.8248
	rs17138331	G	A	0.1391	0.0295	2.26E-06	-0.0336	0.0540	0.4654
	rs281749	C	T	-0.0799	0.0171	3.17E-06	0.0151	0.0164	0.6442
	rs72791296	C	T	-0.2369	0.0466	3.78E-07	-0.0908	0.3629	0.1975
	rs72799710	C	T	0.1014	0.0218	3.21E-06	-0.0080	0.0058	0.8305
	rs74810984	C	T	-0.2206	0.0474	1.96E-06	-0.0672	0.0892	0.5487
	rs76582507	G	A	-0.3175	0.0677	3.26E-06	0.1497	0.5384	0.2190
	rs76583883	G	T	0.2317	0.0511	4.99E-06	0.0640	0.1228	0.3977
	rs76776296	G	A	-0.2997	0.0598	5.55E-07	0.0050	0.0026	0.9455
RANTES									
	rs112072646	G	A	-0.4286	0.0862	6.48E-07	-0.0040	0.0019	0.9636
	rs147509526	C	T	0.358	0.0717	6.93E-07	0.1031	0.1831	0.4269
	rs2251660	C	A	-0.1829	0.0359	3.83E-07	0.0630	0.4023	0.1244
	rs4940620	G	A	0.2494	0.054	3.54E-06	-0.0481	0.0798	0.4539
	rs62438851	G	A	0.1957	0.0414	2.33E-06	0.0898	2.0503	0.0350
	rs7000423	C	T	0.1318	0.0253	1.82E-07	-0.0212	0.0307	0.5112
	rs72793342	G	A	0.1487	0.0308	1.48E-06	0.0294	0.0512	0.4344
	rs74472919	C	T	-0.3313	0.0605	3.97E-08	0.0715	0.1330	0.4091
	rs75613039	C	T	-0.37	0.081	4.81E-06	0.0683	0.1169	0.4408
	rs818452	C	T	-0.2381	0.0505	2.36E-06	0.0587	0.1464	0.3116
SDF1a									
	rs10474392	G	A	-0.0962	0.0178	1.24E-06	-0.0344	0.0901	0.2976
	rs12407262	G	A	-0.1179	0.0266	3.99E-06	0.0161	0.0155	0.7010

	rs4581824	G	T	0.0701	0.0173	3.05E-06	-0.0060	0.0043	0.8386
	rs482700	G	A	0.0893	0.0203	1.57E-06	0.0402	0.1341	0.2355
bNGF									
	rs28637706	G	T	0.1589	0.0263	1.42E-09	-0.0080	0.0062	0.7984
	rs67476890	C	T	-0.1769	0.0379	3.13E-06	0.0020	0.0010	0.9557
	rs71641308	C	T	-0.2043	0.0432	2.30E-06	-0.0545	0.1312	0.3221
	rs72780728	G	A	-0.1883	0.0403	2.99E-06	-0.0060	0.0036	0.9078
	rs73472576	C	T	0.1181	0.0252	2.69E-06	0.0377	0.1347	0.2205
	rs7970581	G	T	-0.138	0.0282	9.27E-07	-0.0237	0.0353	0.4980
	rs9436119	G	A	0.1121	0.0246	3.91E-06	0.0576	0.7702	0.0596
FGFBasic									
	rs13412535	G	A	0.1112	0.0225	7.35E-07	-0.0139	0.0271	0.6072
	rs145577605	G	A	-0.2081	0.0428	9.64E-07	0.0444	0.0585	0.4475
	rs747334	G	A	-0.0751	0.0164	4.53E-06	0.0389	0.0216	0.0725
	rs75168112	C	T	0.1001	0.0214	3.00E-06	0.0213	0.0273	0.4354
	rs9907295	C	T	0.1319	0.0269	7.95E-07	-0.0098	0.0352	0.7810
GCSF									
	rs115256310	G	A	0.6821	0.136	6.73E-07	0.0040	0.0018	0.9761
	rs11903143	G	A	-0.087	0.0176	6.35E-07	-0.0429	0.1667	0.2031
	rs147128865	C	T	-0.27	0.0587	4.92E-06	-0.2013	1.8804	0.0853
	rs1817411	C	T	-0.089	0.0191	3.10E-06	0.0040	0.0024	0.9057
	rs2671444	G	A	0.0784	0.0166	2.48E-06	-0.0141	0.0154	0.6397
	rs74148555	C	T	0.3715	0.0755	1.55E-06	-0.0980	0.3075	0.2501
	rs76287671	C	T	-0.0938	0.0189	6.92E-07	0.0419	0.1223	0.2680
	rs77318030	C	T	0.2045	0.0428	2.21E-06	0.0677	0.1791	0.2944
HGF									
	rs11060254	G	A	0.08	0.0167	1.58E-06	0.0030	0.0017	0.9141
	rs150322232	G	A	-0.2104	0.0463	4.89E-06	0.0159	0.0105	0.8682
	rs1698249	C	A	0.1698	0.0372	4.09E-06	-0.0243	0.0264	0.6429
	rs2003620	C	T	-0.2279	0.0489	2.83E-06	0.0471	0.0744	0.4732
	rs3748034	G	T	-0.1495	0.0234	1.81E-10	-0.0080	0.0056	0.8423
	rs5745687	C	T	0.3072	0.0406	2.75E-14	-0.0296	0.0336	0.6211
	rs62481625	C	T	-0.1091	0.0225	1.18E-06	-0.0545	0.3468	0.1248
MCSF									
	rs116274860	G	T	-0.819	0.1741	2.74E-06	-0.0502	0.0511	0.6743
	rs117867915	C	T	-0.5272	0.1098	1.61E-06	0.0714	0.0803	0.6260
	rs12962919	C	T	-0.3052	0.0662	4.65E-06	-0.0010	0.0004	0.9862
	rs145778765	C	T	0.7993	0.1689	2.20E-06	-0.1972	3.6605	0.0430
	rs56367447	C	T	0.4967	0.0883	1.72E-08	0.0151	0.0106	0.8461
	rs62294910	G	A	-0.3431	0.0691	6.82E-07	-0.1222	1.9552	0.0498
	rs78296352	G	T	-0.527	0.1112	1.05E-06	-0.0257	0.0228	0.7394
	rs9387100	C	T	0.1352	0.0292	4.07E-06	-0.0080	0.0061	0.8096
PDGFbb									
	rs11766649	G	A	-0.0908	0.0196	3.53E-06	-0.0336	0.0785	0.3309
	rs11916118	G	A	-0.0889	0.0194	4.93E-06	-0.0101	0.0075	0.8220
	rs12289510	G	A	0.078	0.0158	7.69E-07	0.0010	0.0004	0.9841
	rs13412535	G	A	-0.3352	0.0214	2.46E-55	-0.0080	0.0059	0.8251
	rs192607922	G	T	0.244	0.0498	1.16E-06	0.1398	0.3559	0.3055
	rs2324229	C	T	-0.0894	0.0161	3.48E-08	0.0070	0.0053	0.8154
	rs28406863	G	T	0.2089	0.0382	4.78E-08	-0.0060	0.0032	0.9410
	rs73162807	C	A	0.2391	0.0499	1.74E-06	-0.1017	0.2626	0.3013

	rs9806745	C	A	-0.1162	0.0163	1.10E-12	-0.0131	0.0128	0.6922
	rs9936075	G	A	0.0782	0.0164	1.76E-06	0.0266	0.0522	0.3900
	rs9941733	G	A	-0.1161	0.0228	3.31E-07	0.0488	0.1809	0.2126
SCF									
	rs113127926	C	A	-0.1982	0.042	2.27E-06	-0.0686	0.2279	0.2366
	rs13412535	G	A	0.1067	0.0213	6.04E-07	-0.0080	0.0059	0.8251
	rs1557570	G	T	-0.1186	0.017	2.74E-12	-0.0198	0.0278	0.5233
	rs1568119	C	T	0.5906	0.1129	1.24E-07	-0.0344	0.0252	0.8285
	rs1942355	C	T	0.0716	0.0157	4.70E-06	0.0667	2.0647	0.0258
	rs4841899	C	T	0.1004	0.0178	1.78E-08	0.0198	0.0271	0.5345
	rs78666213	G	T	0.2744	0.0576	2.59E-06	-0.1290	0.7459	0.1373
	rs80271436	G	A	0.237	0.0485	9.95E-07	-0.0639	0.1453	0.3400
SCGFb									
	rs112346514	C	T	0.3314	0.0711	2.37E-06	0.0070	0.0038	0.9354
	rs116924815	C	T	-0.6079	0.0738	1.74E-16	0.0429	0.0475	0.6340
	rs117716477	C	A	-0.8384	0.0841	1.34E-23	0.2132	1.4994	0.1131
	rs12480722	C	T	-0.1624	0.0355	4.72E-06	0.0139	0.0117	0.7666
	rs139413256	G	A	0.5377	0.1084	7.04E-07	-0.0545	0.0848	0.4797
	rs143829871	C	T	0.1902	0.04	1.90E-06	0.0237	0.0233	0.6922
	rs151194174	G	A	-0.4635	0.0942	1.13E-06	0.0769	0.1213	0.4738
	rs17876031	G	A	0.1514	0.0255	2.25E-09	0.0233	0.0370	0.4704
	rs264162	G	A	-0.1097	0.0234	2.69E-06	0.0131	0.0135	0.6691
	rs4656185	G	A	-0.205	0.0256	1.16E-15	-0.0257	0.0467	0.4172
	rs4737732	G	A	0.1147	0.0252	4.68E-06	-0.0202	0.0256	0.5693
	rs73185877	G	A	-0.5249	0.0711	1.18E-13	0.0587	0.0770	0.5539
	rs7762066	C	T	-0.1389	0.0299	3.50E-06	0.0080	0.0060	0.8138
	rs78217154	C	T	-0.3997	0.0864	3.77E-06	-0.0294	0.0240	0.7798
VEGF									
	rs10153304	G	A	-0.1547	0.0325	1.94E-06	0.0545	0.1625	0.2625
	rs114773511	C	T	-0.2186	0.0442	1.25E-06	-0.3149	0.0284	0.7324
	rs3025021	C	T	-0.0985	0.0203	1.40E-06	0.0336	0.0846	0.3083
	rs34881325	C	T	0.1082	0.0189	1.04E-08	0.0111	0.0103	0.7183
	rs4082730	G	A	-0.2522	0.0534	2.64E-06	0.0513	0.0693	0.5405
	rs4507572	C	T	-0.1007	0.0171	3.34E-09	0.0501	0.0034	0.8617
	rs62401205	C	A	0.2039	0.0412	1.77E-06	0.0954	0.3228	0.2324
	rs67798973	G	A	-0.1389	0.0175	1.29E-15	-0.1440	0.2404	0.1453
	rs6921438	G	A	0.49	0.0175	2.09E-171	-0.0400	0.0005	0.9627
IL1b									
	rs143319329	C	T	-0.2801	0.0715	2.00E-06	0.0387	0.0385	0.6856
	rs1942793	G	T	-0.0717	0.0187	4.98E-06	-0.0478	0.3345	0.1137
	rs4786740	C	A	-0.0845	0.0202	4.67E-06	0.0305	0.0736	0.3211
	rs61335305	C	A	-0.2966	0.0724	1.90E-06	-0.0109	0.0062	0.9246
	rs62015704	G	A	-0.1082	0.0283	2.09E-06	0.0169	0.0162	0.7010
	rs9898641	C	T	0.2032	0.0454	3.59E-06	0.0169	0.0204	0.5909
IL1RA									
	rs12051139	C	T	0.1131	0.0247	4.76E-06	-0.0274	0.0556	0.3777
	rs13412535	G	A	-0.1764	0.0332	1.18E-07	-0.0080	0.0059	0.8251
	rs170117	C	T	0.1617	0.0349	3.87E-06	-0.0402	0.0865	0.3578
	rs2807544	G	A	-0.1175	0.0253	3.41E-06	-0.0237	0.0408	0.4387
	rs4634519	G	A	0.1261	0.0269	2.77E-06	0.0354	0.1007	0.2746
	rs61335305	C	A	-0.4514	0.0918	7.32E-07	-0.0109	0.0062	0.9246

	rs62124990	G	T	0.6961	0.1495	3.22E-06	-0.0526	0.0660	0.5742
	rs7615304	G	A	0.1172	0.0242	1.21E-06	-0.0373	0.1300	0.2259
	rs80336398	C	T	-0.4001	0.0858	2.82E-06	0.0450	0.0411	0.7262
IL2									
	rs12051139	C	T	0.1131	0.0247	4.76E-06	-0.0274	0.0196	0.3777
	rs13412535	G	A	-0.1764	0.0332	1.18E-07	-0.0080	0.0129	0.8251
	rs170117	C	T	0.1617	0.0349	3.87E-06	-0.0402	0.0280	0.3578
	rs2807544	G	A	-0.1175	0.0253	3.41E-06	-0.0237	0.0185	0.4387
	rs4634519	G	A	0.1261	0.0269	2.77E-06	0.0354	0.0220	0.2746
	rs61335305	C	A	-0.4514	0.0918	7.32E-07	-0.0109	0.0276	0.9246
	rs62124990	G	T	0.6961	0.1495	3.22E-06	-0.0526	0.0499	0.5742
	rs7615304	G	A	0.1172	0.0242	1.21E-06	-0.0373	0.0216	0.2259
	rs7806875	T	A	-0.1192	0.0248	1.47E-06	-0.0202	0.0171	0.4978
	rs80336398	C	T	-0.4001	0.0858	2.82E-06	0.0450	0.0562	0.7262
IL2ra									
	rs10110247	G	A	0.1337	0.0293	4.53E-06	-0.0296	0.0460	0.4800
	rs10490280	C	T	-0.1396	0.0304	3.59E-06	-0.0202	0.0255	0.5713
	rs115360066	G	A	-0.1867	0.0379	8.06E-07	-0.0356	0.0573	0.4661
	rs117244812	G	A	0.7064	0.1488	2.10E-06	-0.1345	0.3407	0.3071
	rs6881097	G	A	0.1145	0.0251	4.83E-06	0.0050	0.0032	0.8865
	rs7678976	G	A	-0.3296	0.0715	4.04E-06	-0.0695	0.1963	0.2767
IL4									
	rs10512267	C	T	0.0824	0.0161	2.94E-07	-0.0263	0.0501	0.4011
	rs116705532	G	T	0.4678	0.0978	1.76E-06	0.2319	5.8586	0.0316
	rs117146485	C	T	0.2924	0.0629	2.71E-06	-0.0823	0.1008	0.5860
	rs17713451	G	A	-0.1274	0.0253	4.97E-07	0.0694	0.4695	0.1174
	rs73023729	G	A	0.1796	0.0366	9.03E-07	0.4339	252.8476	0.0014
	rs7613691	G	A	-0.1775	0.0384	4.05E-06	-0.0440	0.0685	0.4788
	rs79597994	C	T	0.5831	0.127	4.32E-06	0.0683	0.1064	0.4790
	rs9508291	C	T	0.1676	0.0359	3.03E-06	-0.0587	0.1367	0.3323
	rs9941733	G	A	-0.114	0.0229	6.88E-07	0.0488	0.1809	0.2126
IL5									
	rs11680908	G	A	-0.2634	0.0554	0.00000203	0.0266	0.0277	0.6639
	rs72831687	G	A	0.5239	0.1109	0.000001691	0.0050	0.0022	0.9775
	rs73040130	C	T	-0.2638	0.0529	6.004E-07	0.0459	0.0753	0.4581
	rs7767396	G	A	-0.1515	0.0246	7.69E-10	-0.0060	0.0042	0.8433
IL6									
	rs1333040	C	T	-0.0738	0.0158	3.17E-06	-0.0161	0.0196	0.5904
	rs13412535	G	A	0.1164	0.0215	7.34E-08	-0.0080	0.0059	0.8251
	rs73273528	C	T	-0.2672	0.0553	9.58E-07	-0.1044	0.4119	0.2000
	rs76856708	C	T	-0.3289	0.07	2.61E-06	-0.0834	0.2164	0.3000
IL7									
	rs141425475	C	T	0.4781	0.1016	2.53E-06	0.0583	0.0823	0.5213
	rs17091524	C	T	-0.4924	0.1013	1.91E-06	-0.1210	0.5145	0.1860
	rs218247	G	C	0.1352	0.0286	2.31E-06	-0.0070	0.0049	0.8491
	rs28793375	C	T	-0.1638	0.0361	4.46E-06	0.0367	0.0660	0.4215
	rs4320361	G	T	0.3245	0.0249	6.87E-39	0.0040	0.0026	0.8816
	rs75904417	C	A	0.1698	0.0349	1.16E-06	0.0305	0.0429	0.5234
	rs77981494	C	T	0.5178	0.1064	1.07E-06	0.1467	0.8105	0.1436
	rs78346957	G	A	-0.4588	0.1007	4.51E-06	-0.0218	0.0155	0.8409
	rs8175379	T	A	-0.1307	0.0285	4.38E-06	0.0030	0.0016	0.9330

	rs9296421	G	T	-0.169	0.0355	2.10E-06	0.0305	0.0418	0.5344
IL8									
	rs11634944	C	T	0.1214	0.0252	1.29E-06	-0.0294	0.0669	0.3398
	rs12075	G	A	-0.12	0.0236	3.88E-07	-0.0315	0.0846	0.2903
	rs141926526	C	A	0.6149	0.1308	2.57E-06	0.0050	0.0025	0.9497
	rs2673604	C	A	0.1266	0.0255	7.02E-07	0.0198	0.0265	0.5453
IL9									
	rs41294750	C	T	-0.3514	0.0748	2.37E-06	-0.0169	0.0117	0.8495
	rs4880409	C	T	0.3355	0.0723	3.50E-06	-0.0910	0.2093	0.3364
	rs61867538	C	T	-0.3566	0.0774	3.93E-06	-0.1398	1.7236	0.0646
	rs7232268	G	A	-0.2759	0.0587	2.53E-06	0.0060	0.0032	0.9400
	rs7242404	G	A	0.1228	0.0264	3.27E-06	-0.0040	0.0023	0.9127
	rs76963786	C	T	0.2865	0.0557	4.50E-07	-0.0169	0.0149	0.7422
IL10									
	rs10457128	G	A	0.0865	0.0172	5.24E-07	-0.0080	0.0064	0.7870
	rs10493718	C	A	0.11	0.0222	7.16E-07	-0.0247	0.0377	0.4870
	rs11206302	C	T	0.1189	0.0251	2.20E-06	0.0325	0.0421	0.5596
	rs2086656	C	T	0.0789	0.0171	3.78E-06	0.0431	0.1822	0.1869
	rs282258	C	T	-0.0992	0.0162	1.00E-09	-0.0315	0.0823	0.2982
	rs3025021	C	T	-0.0947	0.0195	1.46E-06	0.0336	0.0846	0.3083
	rs4349809	G	T	-0.2853	0.0165	5.77E-67	-0.0090	0.0076	0.7658
	rs465757	G	A	-0.084	0.0174	1.17E-06	0.0497	0.3270	0.1209
	rs6085948	G	A	-0.098	0.0202	1.25E-06	0.0178	0.0208	0.6086
	rs6458375	C	T	-0.0946	0.0198	2.07E-06	0.0492	0.2305	0.1690
	rs7088799	G	T	0.0852	0.0167	3.23E-07	-0.0040	0.0025	0.8840
IL12									
	rs12199215	C	T	-0.1278	0.0192	5.11E-11	-0.0315	0.0709	0.3433
	rs145023524	G	A	-0.279	0.0394	1.54E-12	0.1543	0.2878	0.4081
	rs17229494	G	A	0.1172	0.0257	4.93E-06	-0.0040	0.0021	0.9400
	rs282258	C	T	-0.073	0.0156	3.21E-06	-0.0315	0.0823	0.2982
	rs3025021	C	T	-0.0898	0.0188	2.20E-06	0.0336	0.0846	0.3083
	rs4349809	G	T	-0.3777	0.0159	2.56E-124	-0.0090	0.0076	0.7658
	rs71361173	G	T	-0.111	0.0239	3.06E-06	-0.0151	0.0140	0.7201
	rs7754905	G	A	0.1029	0.019	4.28E-08	-0.0398	0.1253	0.2491
	rs7757246	C	T	-0.139	0.0285	1.97E-06	0.0573	0.2054	0.2199
	rs782107	G	A	-0.075	0.0156	1.60E-06	0.0119	0.0116	0.6946
	rs79121401	C	T	-0.5548	0.1206	4.24E-06	0.1765	1.1373	0.1233
IL13									
	rs117795020	G	A	0.3522	0.0716	9.86E-07	-0.0040	0.0018	0.9724
	rs12623722	G	A	0.1185	0.0258	4.19E-06	0.0367	0.1109	0.2591
	rs139083458	C	T	-0.9902	0.2107	2.81E-06	0.1165	0.2804	0.3223
	rs2370048	G	A	0.1235	0.0266	3.35E-06	-0.0387	0.1214	0.2503
	rs6799107	C	T	0.1459	0.0301	1.25E-06	0.0421	0.1329	0.2486
	rs7073807	C	T	-0.1682	0.0356	2.37E-06	-0.0554	0.1876	0.2324
	rs75383097	G	C	0.536	0.1165	4.29E-06	0.1602	0.5485	0.2297
	rs75438658	C	T	0.343	0.0625	4.12E-08	-0.1985	0.7900	0.1983
	rs75995699	G	A	-0.3319	0.0698	2.64E-06	-0.1275	0.5960	0.1694
	rs76339001	T	A	0.4313	0.0886	1.15E-06	-0.0943	0.2273	0.3218
	rs7757246	C	T	-0.2171	0.0423	3.53E-07	0.0573	0.2054	0.2199
	rs9296421	G	T	-0.1746	0.0349	6.30E-07	0.0305	0.0418	0.5344
	rs9472168	G	A	-0.4244	0.0248	1.08E-65	-0.0060	0.0044	0.8315

IL16								
rs117217798	C	T	0.2036	0.0444	4.15E-06	0.0823	0.3593	0.1812
rs117916513	G	A	0.502	0.0986	3.79E-07	0.1462	0.3787	0.3005
rs1255143	C	T	-0.1306	0.0242	7.10E-08	0.0060	0.0042	0.8435
rs12765671	G	A	0.6023	0.1318	4.84E-06	-0.0227	0.0174	0.8084
rs144691581	G	A	-0.4882	0.0967	4.20E-07	0.0274	0.0201	0.8272
rs1801020	G	A	-0.1733	0.0272	4.53E-10	-0.0050	0.0032	0.8843
rs4253283	C	T	-0.146	0.0262	1.75E-08	0.0090	0.0074	0.7792
rs4513633	C	A	0.2239	0.0453	7.44E-07	0.0315	0.0495	0.4758
rs4778636	G	A	0.7272	0.0633	1.11E-30	0.0387	0.0650	0.4487
rs9706053	C	T	-0.4582	0.0932	7.01E-07	-0.0315	0.0272	0.7532
IL17								
rs117029961	G	A	-0.4585	0.1015	4.94E-06	0.0747	0.0821	0.6375
rs117556572	C	T	0.5102	0.1099	3.28E-06	0.0141	0.0086	0.8978
rs1530455	C	T	-0.108	0.0173	4.87E-10	0.0161	0.0194	0.5947
rs17106604	C	T	-0.1129	0.0225	6.37E-07	-0.0573	0.2101	0.2151
rs17282552	C	T	0.2001	0.0405	8.21E-07	-0.0060	0.0031	0.9461
rs184080173	C	T	-0.2384	0.0471	4.19E-07	-0.0758	0.2808	0.2128
rs187475560	C	T	0.2434	0.052	3.29E-06	0.1543	0.3575	0.3340
rs62191444	G	T	0.1136	0.0247	4.22E-06	-0.0545	0.2561	0.1685
rs78296352	G	T	-0.3027	0.0646	4.27E-06	-0.0257	0.0228	0.7394
rs78612928	C	T	-0.1037	0.0222	2.62E-06	-0.0377	0.0898	0.3253
IL18								
rs10414578	C	T	0.1771	0.035	4.16E-07	-0.0602	0.2401	0.1978
rs115267715	C	T	-0.4508	0.08	1.72E-08	-0.1424	0.3120	0.3518
rs116383510	C	A	0.5426	0.1056	3.00E-07	0.1415	0.4398	0.2524
rs116656892	C	T	-0.5298	0.0925	1.05E-08	-0.0672	0.1062	0.4730
rs11700536	C	T	-0.1156	0.025	4.21E-06	0.0070	0.0052	0.8213
rs117266781	C	T	-0.6841	0.1468	3.15E-06	0.2408	1.9273	0.0994
rs144841621	C	T	-0.518	0.1141	3.81E-06	-0.2151	0.5259	0.3175
rs17229943	C	A	0.312	0.0463	1.62E-11	0.0325	0.0366	0.6248
rs1852105	C	T	-0.3036	0.0661	4.32E-06	-0.0159	0.0122	0.8080
rs1979967	C	T	-0.1402	0.0286	9.45E-07	0.0356	0.0851	0.3244
rs2729385	G	A	-0.1231	0.0262	3.79E-06	0.0263	0.0490	0.4095
rs385076	C	T	0.2432	0.0248	1.66E-22	-0.0050	0.0034	0.8617
rs4482818	G	A	-0.1286	0.0244	1.45E-07	-0.0253	0.0472	0.4084
rs658805	G	A	-0.1226	0.0244	4.94E-07	-0.0080	0.0060	0.8131
rs71478720	C	T	0.2669	0.0276	3.71E-22	-0.0109	0.0094	0.7560
rs78623212	C	T	-0.8705	0.1778	6.71E-07	-0.1115	0.3833	0.2290
rs78716465	G	A	-0.3265	0.0682	1.63E-06	-0.1249	1.0455	0.0951
IFNg								
rs10487554	G	A	0.0895	0.0183	1.09E-06	-0.0440	0.1919	0.1814
rs112783231	G	A	0.2408	0.0511	1.96E-06	-0.0178	0.0119	0.8677
rs115729819	G	A	-0.2484	0.0515	1.38E-06	-0.2058	1.3395	0.1221
rs11843756	G	T	-0.184	0.0393	3.09E-06	0.1544	1.4517	0.0847
rs12420286	C	T	-0.2376	0.0501	2.08E-06	0.0953	0.4261	0.1770
rs1867282	C	T	-0.0774	0.0166	3.15E-06	0.0325	0.0799	0.3162
rs2073438	G	A	-0.0898	0.0188	1.68E-06	0.0171	0.0202	0.6039
rs74148555	C	T	0.3732	0.0774	2.64E-06	-0.0980	0.3075	0.2501
rs78296352	G	T	-0.343	0.0652	1.38E-07	-0.0257	0.0228	0.7394
MIF								

	rs113218956	G	A	0.8948	0.1879	2.26E-06	-0.1035	0.1097	0.6544
	rs118055855	C	T	-0.6907	0.15	4.13E-06	0.1380	0.4608	0.2354
	rs12594190	G	A	-0.1355	0.0267	3.70E-07	0.0060	0.0039	0.8762
	rs13142904	C	T	0.223	0.0425	2.56E-07	0.0263	0.0280	0.6527
	rs141009259	C	T	0.6178	0.1322	2.47E-06	-0.2132	1.0592	0.1595
	rs78098071	C	T	0.4867	0.0918	1.78E-07	0.0402	0.0336	0.7680
TNFa									
	rs10834997	G	A	0.1247	0.0258	1.33E-06	0.0119	0.0110	0.7216
	rs111332265	G	A	0.3766	0.0754	6.63E-07	-0.0694	0.1806	0.2991
	rs115669577	G	A	-0.9889	0.1995	8.28E-07	-0.2708	3.8038	0.0568
	rs79105320	G	A	-0.5605	0.1179	3.59E-06	-0.1213	0.3492	0.2718
	rs8121916	C	A	-0.1306	0.0278	2.72E-06	-0.0402	0.1304	0.2421
TNFb									
	rs10925040	C	T	-0.1755	0.0373	2.67E-06	0.0315	0.0798	0.3069
	rs116196280	G	T	-0.7179	0.1006	4.98E-13	0.2021	2.3998	0.0671
	rs148867298	C	T	-0.3157	0.069	4.52E-06	0.0812	0.3726	0.1725
	rs753274	C	T	0.1736	0.0371	2.77E-06	0.0010	0.0004	0.9841
	rs7629875	G	A	-0.3766	0.0774	1.37E-06	0.0825	0.3036	0.2142
	rs78296352	G	T	-1.2215	0.1366	4.76E-21	-0.0257	0.0228	0.7394
TRAIL									
	rs10164260	G	A	-0.1018	0.0211	1.51E-06	0.1143	6.2464	0.0146
	rs11081739	G	A	-0.1411	0.0202	3.34E-12	-0.0237	0.0324	0.5355
	rs11618126	G	A	-0.8908	0.1914	1.46E-06	-0.1335	0.3695	0.2822
	rs11657269	G	A	-0.1188	0.026	4.78E-06	0.0198	0.0220	0.6316
	rs11699445	G	T	-0.0746	0.0161	3.27E-06	0.0060	0.0042	0.8409
	rs13185784	G	A	-0.0846	0.0183	3.90E-06	-0.0050	0.0033	0.8740
	rs13278062	G	T	-0.0801	0.0157	3.57E-07	-0.0070	0.0054	0.8092
	rs146783010	G	A	0.6016	0.135	4.83E-06	-0.0683	0.0868	0.5683
	rs148051545	C	T	0.3921	0.0848	3.86E-06	-0.1089	0.3674	0.2330
	rs193112415	C	T	1.0421	0.0623	2.15E-62	0.0526	0.0597	0.6219
	rs3136596	G	A	-0.1147	0.0209	3.65E-08	0.0161	0.0168	0.6617

Abbreviations: log(OR): log odds ratio; se: standard error a Outliers detected by MR-PRESSO were removed. Beta for inflammatory cytokines represent change in standard deviation per 1 copy of effect allele.

Supplementary Table S10.The reverse MR estimates for Dataset 2.

Category	Exposure	No. of SNPs	IVW			MR-Lasso			MR-Egger			Weighted Median			Simple Mode			Weighted Mode		
			OR	95% CI	pval	OR	95% CI	pval	OR	95% CI	pval	OR	95% CI	pval	OR	95% CI	pval	OR	95% CI	pval
**Chemokines**																				
	CTACK	4	1.024	(0.936,1.120)	0.607	1.047	(0.936,1.120)	0.607	0.875	(0.319,2.399)	0.820	1.019	(0.920,1.128)	0.722	1.010	(0.889,1.146)	0.893	1.018	(0.918,1.129)	0.759
	Eotaxin	4	1.025	(0.965,1.089)	0.418	1.031	(0.966,1.089)	0.418	0.898	(0.459,1.756)	0.782	1.021	(0.954,1.091)	0.551	1.015	(0.933,1.105)	0.747	1.017	(0.950,1.088)	0.657
	GROa	4	0.937	(0.855,1.027)	0.162	1.048	(0.855,1.026)	0.162	0.748	(0.267,2.094)	0.636	0.928	(0.838,1.027)	0.149	0.920	(0.799,1.058)	0.326	0.927	(0.831,1.033)	0.265
	IP10	4	1.022	(0.926,1.128)	0.662	1.051	(0.927,1.127)	0.662	0.827	(0.221,3.099)	0.805	1.012	(0.916,1.117)	0.817	1.000	(0.894,1.119)	0.999	1.011	(0.910,1.123)	0.854
	MCP1	4	0.983	(0.926,1.044)	0.577	1.031	(0.926,1.044)	0.577	0.583	(0.299,1.136)	0.254	0.966	(0.902,1.033)	0.311	0.975	(0.888,1.070)	0.626	0.964	(0.902,1.031)	0.366
	MCP3	4	1.157	(0.985,1.360)	0.075	1.085	(0.985,1.359)	0.075	1.362	(0.227,8.172)	0.768	1.158	(0.971,1.382)	0.102	1.177	(0.933,1.485)	0.263	1.169	(0.957,1.428)	0.224
	MIG	4	1.012	(0.926,1.107)	0.786	1.046	(0.926,1.106)	0.786	0.750	(0.245,2.299)	0.665	1.002	(0.907,1.106)	0.971	0.990	(0.873,1.122)	0.883	1.001	(0.911,1.099)	0.992
	MIP1a	4	0.913	(0.832,1.002)	0.056	1.048	(0.832,1.002)	0.056	1.122	(0.322,3.904)	0.873	0.918	(0.821,1.027)	0.135	0.895	(0.768,1.042)	0.249	0.913	(0.812,1.026)	0.223
	MIP1b	4	1.099	(1.035,1.166)	0.002	1.030	(1.036,1.166)	0.002	1.257	(0.569,2.780)	0.628	1.107	(1.033,1.186)	0.004	1.123	(1.026,1.229)	0.086	1.108	(1.028,1.195)	0.076
	RANTES	4	0.957	(0.870,1.051)	0.356	1.049	(0.870,1.051)	0.356	0.455	(0.161,1.289)	0.276	0.929	(0.840,1.028)	0.156	0.928	(0.795,1.083)	0.411	0.923	(0.823,1.034)	0.261
	SDF1a	4	1.051	(0.988,1.117)	0.116	1.031	(0.988,1.117)	0.116	0.951	(0.478,1.894)	0.900	1.045	(0.976,1.120)	0.206	1.061	(0.965,1.165)	0.309	1.036	(0.962,1.115)	0.423
**Growthfactors**																				
	bNGF	4	1.084	(0.989,1.188)	0.085	1.048	(0.989,1.188)	0.085	0.904	(0.323,2.525)	0.865	1.101	(0.993,1.221)	0.068	1.104	(0.972,1.254)	0.225	1.103	(0.987,1.234)	0.183
	FGFBasic	4	1.013	(0.951,1.078)	0.692	1.033	(0.951,1.078)	0.692	1.067	(0.523,2.181)	0.874	1.011	(0.939,1.089)	0.769	0.955	(0.854,1.068)	0.481	0.985	(0.904,1.074)	0.759
	GCSF	4	0.983	(0.925,1.044)	0.575	1.031	(0.925,1.044)	0.575	0.918	(0.465,1.810)	0.827	0.985	(0.920,1.055)	0.662	1.001	(0.913,1.098)	0.985	0.985	(0.918,1.057)	0.710
	HGF	4	1.071	(1.009,1.136)	0.025	1.030	(1.009,1.137)	0.025	0.712	(0.366,1.385)	0.423	1.061	(0.992,1.134)	0.084	1.073	(0.977,1.179)	0.236	1.058	(0.984,1.137)	0.227
	MCSF	4	0.987	(0.885,1.102)	0.822	1.058	(0.884,1.102)	0.822	1.039	(0.304,3.551)	0.957	0.998	(0.880,1.132)	0.978	1.025	(0.872,1.205)	0.785	1.000	(0.879,1.137)	0.995
	PDGFbb	4	0.991	(0.934,1.052)	0.767	1.030	(0.933,1.052)	0.767	0.715	(0.368,1.391)	0.428	0.987	(0.917,1.062)	0.720	0.963	(0.867,1.069)	0.528	0.970	(0.894,1.053)	0.519
	SCF	4	1.044	(0.944,1.155)	0.400	1.053	(0.944,1.155)	0.400	1.406	(0.377,5.249)	0.663	1.058	(0.992,1.129)	0.088	1.076	(0.979,1.182)	0.228	1.061	(0.992,1.134)	0.182
	SCGFb	4	1.009	(0.923,1.103)	0.848	1.046	(0.923,1.103)	0.848	0.523	(0.192,1.421)	0.332	1.003	(0.904,1.112)	0.962	1.083	(0.922,1.272)	0.405	0.992	(0.887,1.108)	0.891
	VEGF	4	0.981	(0.920,1.046)	0.559	1.034	(0.919,1.046)	0.559	0.775	(0.376,1.599)	0.562	0.976	(0.910,1.048)	0.506	0.977	(0.884,1.079)	0.673	0.971	(0.902,1.046)	0.492
**Interleukins**																				
	IL1b	4	0.944	(0.873,1.022)	0.157	1.041	(0.873,1.022)	0.157	0.777	(0.272,2.220)	0.684	0.945	(0.871,1.026)	0.177	0.967	(0.860,1.089)	0.620	0.946	(0.865,1.034)	0.306

	IL1RA	4	0.931	(0.851,1.018)	0.116	1.047	(0.850,1.018)	0.116	0.640	(0.233,1.753)	0.477	0.931	(0.840,1.032)	0.172	0.968	(0.844,1.109)	0.670	0.903	(0.804,1.014)	0.183
	IL2	4	0.990	(0.903,1.086)	0.835	1.048	(0.903,1.085)	0.835	0.517	(0.184,1.451)	0.337	0.984	(0.885,1.095)	0.772	1.034	(0.890,1.202)	0.690	0.977	(0.871,1.095)	0.716
	IL2ra	4	0.914	(0.836,1.000)	0.049	1.047	(0.836,1.000)	0.049	0.839	(0.307,2.288)	0.764	0.908	(0.821,1.004)	0.061	0.898	(0.795,1.015)	0.184	0.908	(0.817,1.009)	0.172
	IL4	4	1.016	(0.957,1.080)	0.597	1.031	(0.957,1.080)	0.597	0.870	(0.443,1.706)	0.724	1.014	(0.945,1.089)	0.699	1.021	(0.929,1.122)	0.699	0.993	(0.912,1.082)	0.882
	IL5	4	0.898	(0.818,0.986)	0.024	1.049	(0.818,0.986)	0.024	0.440	(0.154,1.257)	0.265	0.872	(0.786,0.968)	0.010	0.887	(0.768,1.025)	0.204	0.872	(0.781,0.975)	0.095
	IL6	4	1.008	(0.949,1.070)	0.799	1.031	(0.949,1.070)	0.799	0.813	(0.416,1.589)	0.606	1.020	(0.950,1.095)	0.584	1.023	(0.931,1.125)	0.669	0.978	(0.899,1.063)	0.637
	IL7	4	0.873	(0.796,0.958)	0.004	1.048	(0.796,0.958)	0.004	0.459	(0.162,1.301)	0.281	0.865	(0.775,0.966)	0.010	0.884	(0.751,1.040)	0.235	0.859	(0.765,0.964)	0.082
	IL8	4	0.975	(0.866,1.097)	0.671	1.062	(0.866,1.096)	0.671	0.328	(0.118,0.913)	0.166	0.943	(0.851,1.045)	0.262	1.245	(1.055,1.471)	0.081	0.937	(0.857,1.025)	0.253
	IL9	4	0.987	(0.903,1.080)	0.781	1.047	(0.903,1.080)	0.781	1.001	(0.366,2.737)	0.999	0.983	(0.890,1.084)	0.728	0.986	(0.864,1.125)	0.846	0.984	(0.884,1.095)	0.788
	IL10	4	0.998	(0.938,1.061)	0.938	1.033	(0.938,1.061)	0.938	0.848	(0.424,1.695)	0.686	0.997	(0.929,1.071)	0.942	0.997	(0.905,1.099)	0.957	0.991	(0.920,1.066)	0.816
	IL12	4	1.029	(0.969,1.092)	0.349	1.030	(0.969,1.092)	0.349	0.992	(0.510,1.929)	0.983	1.038	(0.969,1.113)	0.289	1.044	(0.958,1.138)	0.395	1.032	(0.958,1.111)	0.467
	IL13	4	0.957	(0.873,1.048)	0.339	1.047	(0.873,1.048)	0.339	1.008	(0.362,2.805)	0.989	0.958	(0.868,1.058)	0.397	0.955	(0.835,1.092)	0.546	0.958	(0.862,1.066)	0.490
	IL16	4	1.066	(0.973,1.169)	0.170	1.048	(0.972,1.169)	0.170	1.663	(0.594,4.654)	0.435	1.085	(0.979,1.202)	0.119	1.079	(0.944,1.234)	0.347	1.085	(0.981,1.201)	0.211
	IL17	4	1.020	(0.959,1.085)	0.529	1.033	(0.959,1.085)	0.529	1.068	(0.517,2.209)	0.875	1.031	(0.960,1.108)	0.399	1.040	(0.947,1.142)	0.476	1.024	(0.951,1.102)	0.575
	IL18	4	1.027	(0.939,1.123)	0.564	1.047	(0.939,1.123)	0.564	1.118	(0.408,3.065)	0.848	1.027	(0.929,1.137)	0.599	1.021	(0.894,1.164)	0.782	1.027	(0.924,1.141)	0.659
**others**																				
	IFNg	4	1.025	(0.963,1.090)	0.441	1.033	(0.963,1.090)	0.441	0.946	(0.473,1.894)	0.890	1.025	(0.954,1.101)	0.496	1.011	(0.925,1.105)	0.831	1.010	(0.945,1.080)	0.787
	MIF	4	1.085	(0.990,1.189)	0.080	1.048	(0.990,1.189)	0.080	1.512	(0.540,4.236)	0.514	1.093	(0.988,1.210)	0.085	1.116	(0.966,1.288)	0.233	1.096	(0.981,1.225)	0.203
	TNFa	4	0.975	(0.889,1.070)	0.597	1.048	(0.890,1.069)	0.597	0.525	(0.186,1.482)	0.348	0.941	(0.845,1.047)	0.261	0.939	(0.792,1.114)	0.522	0.941	(0.839,1.055)	0.373
	TNFb	4	0.802	(0.703,0.916)	0.001	1.069	(0.703,0.916)	0.001	0.583	(0.145,2.349)	0.527	0.789	(0.680,0.916)	0.002	0.788	(0.656,0.946)	0.084	0.789	(0.667,0.934)	0.070
	TRAIL	4	0.976	(0.919,1.036)	0.427	1.031	(0.919,1.037)	0.427	1.295	(0.665,2.524)	0.527	0.987	(0.924,1.054)	0.693	0.980	(0.887,1.084)	0.724	0.986	(0.916,1.061)	0.736

Abbreviations: CI, Confidence interval; pval, p-value; SNPs, single nucleotide polymorphisms

OR and 95% CI represent change in SD of Inflammatory regulators per log odds increase in cirrhosis.

After correcting for multiple comparison, p-value < 0.05/41 = 0.0012 was considered as significant.

Supplementary Table S11. Pleiotropy and heterogeneity tests of cirrhosis on forty-one inflammatory cytokines for Dataset 2.

Category	Exposure	Pleiotropy Test				Heterogeneity Test		Recommended Method
		MR Egger			PRESSO	Q1	Q2	
		Intercept	SE	Pval	Pval	Pval	Pval	
**Chemokines**	CTACK	0.060	0.197	0.788	0.884	0.794	0.626	IVW
	Eotaxin	0.051	0.131	0.735	0.940	0.929	0.859	IVW
	GROa	0.087	0.201	0.709	0.815	0.725	0.568	IVW
	IP10	0.081	0.258	0.782	0.512	0.302	0.176	IVW
	MCP1	0.201	0.130	0.263	0.517	0.409	0.774	IVW
	MCP3	-0.062	0.350	0.875	0.994	0.995	0.979	IVW
	MIG	0.115	0.219	0.651	0.609	0.414	0.285	IVW
	MIP1a	-0.079	0.244	0.776	0.483	0.376	0.229	IVW
	MIP1b	-0.052	0.155	0.770	0.592	0.391	0.241	IVW
	RANTES	0.286	0.203	0.295	0.511	0.370	0.557	IVW
	SDF1a	0.038	0.135	0.804	0.930	0.966	0.912	IVW
**Growthfactors**	bNGF	0.070	0.201	0.761	0.961	0.981	0.971	IVW
	FGFBasic	-0.020	0.140	0.898	0.541	0.548	0.350	IVW
	GCSF	0.026	0.133	0.861	0.825	0.786	0.599	IVW
	HGF	0.157	0.130	0.351	0.679	0.576	0.767	IVW
	MCSF	-0.019	0.240	0.943	0.799	0.714	0.508	IVW
	PDGFbb	0.125	0.130	0.437	0.617	0.624	0.661	IVW
	SCF	-0.114	0.257	0.700	0.140	0.346	0.197	IVW
	SCGFb	0.253	0.196	0.325	0.626	0.628	0.966	IVW
	VEGF	0.090	0.142	0.588	0.837	0.856	0.833	IVW
**Interleukins**	IL1b	0.075	0.205	0.749	0.471	0.302	0.181	IVW
	IL1RA	0.144	0.197	0.541	0.827	0.897	0.970	IVW
	IL2	0.250	0.341	0.341	0.863	0.643	0.941	IVW
	IL2ra	0.033	0.196	0.881	0.890	0.841	0.668	IVW
	IL4	0.024	0.024	0.395	0.720	0.879	0.792	IVW
	IL5	-0.101	0.046	0.118	0.299	0.389	0.541	IVW
	IL6	0.083	0.131	0.592	0.736	0.830	0.785	IVW
	IL7	0.247	0.204	0.348	0.660	0.639	0.898	IVW
	IL8	0.419	0.200	0.171	0.290	0.723	0.170	IVW
	IL9	-0.005	0.197	0.981	0.948	0.952	0.843	IVW
	IL10	0.063	0.135	0.689	0.956	0.962	0.963	IVW
	IL12	0.014	0.130	0.923	0.857	0.880	0.719	IVW
	IL13	-0.020	0.200	0.929	0.996	0.994	0.965	IVW
	IL16	-0.171	0.201	0.485	0.825	0.761	0.801	IVW
	IL17	-0.018	0.142	0.911	0.599	0.526	0.330	IVW
	IL18	-0.033	0.197	0.883	0.977	0.959	0.870	IVW
**others**	IFNg	0.031	0.136	0.842	0.921	0.889	0.962	IVW
	MIF	-0.128	0.201	0.591	0.788	0.718	0.624	IVW
	TNFa	0.238	0.203	0.361	0.584	0.678	0.540	IVW
	TNFb	0.122	0.270	0.696	0.948	0.902	0.831	IVW
	TRAIL	-0.109	0.130	0.492	0.594	0.473	0.403	IVW

Q1 pval: p value of Q test from IVW method; Q2 pval: p value of Q test from MR-Egger method Abbreviations: pval, p-value; Q, Cochran Q statistics; SNPs, single nucleotide polymorphisms; IVW, the inverse variance weighted method.

Supplementary Table S12. Details of cirrhosis predicting SNPs with inflammatory cytokines for Dataset 2.

SNP	effect allele	other allele	Log(OR)	EAF	se	pval	F
rs56373884	A	G	0.375	0.156	0.037	6.54E-23	51.082
rs58542926	T	C	0.323	0.074	0.033	4.15E-10	47.788
rs738408	T	C	0.417	0.216	0.052	2.58E-35	32.487
rs79220007	C	T	0.322	0.073	0.031	1.28E-10	55.378

Abbreviations: EAF: effect allele frequency, Log(OR): log odds ratio, se: standard error;

Log(OR) for cirrhosis represents log(OR) change in cirrhosis risk per 1 copy of effect allele

Supplementary Table S13. STROBE-MR checklist of recommended items to address in reports of Mendelian randomization studies[1 2]

Item No.	Section	Checklist item	Page No.	Relevant text from manuscript
1	TITLE and ABSTRACT	Indicate Mendelian randomization (MR) as the study's design in the title and/or the abstract if that is a main purpose of the study	1	The causal relationship between inflammatory cytokines and liver cirrhosis: a bidirectional two-sample Mendelian randomization study and the first conclusions
	INTRODUCTION			
2	Background	Explain the scientific background and rationale for the reported study. What is the exposure? Is a potential causal relationship between exposure and outcome plausible? Justify why MR is a helpful method to address the study question	1	The exposure and outcome of this study were two-way MR studies performed for inflammatory cytokines or cirrhosis. Certain studies have demonstrated that inflammatory factors can have an impact on cirrhosis progression, and there is a lack of evidence that there is an association between the above exposures and outcomes. Therefore, MR is used to investigate whether there is an association between these exposures and outcomes, with the ultimate goal of preventing or delaying disease progression.
3	Objectives	State specific objectives clearly, including pre-specified causal hypotheses (if any). State that MR is a method that, under specific assumptions, intends to estimate causal effects	2	This study performed a two-sample bidirectional magnetic resonance analysis using the Genome-Wide Association Study (GWAS) database to investigate the causal relationship between inflammatory cytokines and cirrhosis.
	METHODS			
4	Study design and data sources	Present key elements of the study design early in the article. Consider including a table listing sources of data for all phases of the study. For each data source contributing to the analysis, describe the following:		
	a)	Setting: Describe the study design and the underlying population, if possible. Describe the setting, locations, and relevant dates, including periods of recruitment, exposure, follow-up, and data collection, when available.	3	The data for 41 circulating inflammatory factors analysis was sourced from a genome-wide meta-analysis of healthy Finnish subjects. The meta-analysis was based on three Finnish cohorts, including the Cardiovascular Risk in Young Finns Study (YFS) and FINRISK 1997 and 2002. The data for cirrhosis analysis was sourced from the OpenGWAS project, which is an online resource developed at the MRC Integrative Epidemiology Unit (IEU) at the University of Bristol and UK Biobank which is a large-scale biomedical database and

				research resource containing de-identified genetic, lifestyle and health information and biological samples.
	b)	Participants: Give the eligibility criteria, and the sources and methods of selection of participants. Report the sample size, and whether any power or sample size calculations were carried out prior to the main analysis	3	Cirrhosis dataset1 comprised 1931 cases and 216,861 controls of European ancestry(https://gwas.mrcieu.ac.uk/datasets/finn-b-CIRRHOSIS_BROAD/). Cirrhosis dataset2 comprised 2,701 cases and 16,206 controls of European ancestry(UKB data is downloaded under the application number :56270). The diagnostic criteria was ICD-10/K70.2 (alcoholic fibrosis and sclerosis of the liver), K70.3 (alcoholic cirrhosis), K70.4 (alcoholic hepatic failure), K74.0 (hepatic fibrosis), K74.1 (hepatic sclerosis), K74.2 (hepatic fibrosis with hepatic sclerosis), K74.6 (other and unspecific cirrhosis of liver), K76.6 (portal hypertension), or I85 (esophageal varices)
	c)	Describe measurement, quality control and selection of genetic variants	3	Selection of instrumental variables (IVs) section
	d)	For each exposure, outcome, and other relevant variables, describe methods of assessment and diagnostic criteria for diseases	3	GWAS data sources section
	e)	Provide details of ethics committee approval and participant informed consent, if relevant	-	
5	Assumptions	Explicitly state the three core IV assumptions for the main analysis (relevance, independence and exclusion restriction) as well assumptions for any additional or sensitivity analysis	2	MR analysis relies on three fundamental assumptions: exclusion restriction, independence, relevance. It is hypothesized that the identified genetic variants are linked to the risk factor (relevance) but not influenced by any confounding factors in the association between the risk factor and the outcome (independence). Furthermore, it is suggested that these variants solely affect the outcome through the specific risk factor of interest (exclusion restriction). In this two-way study, two GWASs were utilized to identify significant single nucleotide polymorphisms (SNPs) associated with 41 inflammatory cytokines and liver cirrhosis. (Figure 1).
6	Statistical methods: main analysis	Describe statistical methods and statistics used		
	a)	Describe how quantitative variables were handled in the analyses (i.e., scale, units, model)	4	Initially, we established a genome-wide significant threshold ($p < 5 \times 10\text{-}8$) for selecting SNPs strongly related to liver cirrhosis and inflammatory cytokines. To address the limited

				available SNPs for cytokines as exposures, cutoff (p <5 × 10-6) was employed.<br>The strength of the instruments was evaluated using the F-statistic to address potential biases arising from weak instruments
	b)	Describe how genetic variants were handled in the analyses and, if applicable, how their weights were selected	4	Furthermore, to mitigate issues related to linkage disequilibrium, the identified SNPs were subjected to clumping (kb = 10,000, r2 = 0.01).
	c)	Describe the MR estimator (e.g. two-stage least squares, Wald ratio) and related statistics. Detail the included covariates and, in case of two-sample MR, whether the same covariate set was used for adjustment in the two samples	3,4	Statistical analyses section
	d)	Explain how missing data were addressed	4	SNPs with an F-value below 10 were deemed weak instruments and were therefore excluded from the analysis. Finally, in instances where SNPs were unavailable in the outcome summary, they were substituted with proxy SNPs sourced from LDlink
	e)	If applicable, indicate how multiple testing was addressed	-	-
7	Assessment of assumptions	Describe any methods or prior knowledge used to assess the assumptions or justify their validity	3	Furthermore, it is suggested that these variants solely affect the outcome through the specific risk factor of interest (exclusion restriction). In this two-way study, two GWASs were utilized to identify significant single nucleotide polymorphisms (SNPs) associated with 41 inflammatory cytokines and liver cirrhosis. (Figure 1).
8	Sensitivity analyses and additional analyses	Describe any sensitivity analyses or additional analyses performed (e.g. comparison of effect estimates from different approaches, independent replication, bias analytic techniques, validation of instruments, simulations)	4	For MR analysis, we first performed heterogeneity and pleiotropy testing. We used the MR-PRESSO outlier test to detect and remove abnormal instrumental variables. The strength of the IVs was evaluated using F statistics, and heterogeneity and horizontal pleiotropy between inflammatory cytokines and cirrhosis were investigated by Cochran's Q test. Heterogeneity was defined as a p-value < 0.05 indicating statistical significance. MR–Egger regression (based on intercept terms) and Mendelian randomization pleiotropy residual sum and outlier (MR-PRESSO) were used to assess horizontal pleiotropy. When the MR–Egger outcome deviated from 0 or its p-value was < 0.05, this indicated the presence

				of horizontal pleiotropy. In addition, we performed sensitivity analyses to detect and correct for pleiotropy in the estimates.
9	Software and pre-registration			
	a)	Name statistical software and package(s), including version and settings used	4	Statistical analyses were performed using specific R packages: TwoSampleMR, MR-PRESSO and MendelianRandomization.
	b)	State whether the study protocol and details were pre-registered (as well as when and where)	-	
	RESULTS			
10	Descriptive data			
	a)	Report the numbers of individuals at each stage of included studies and reasons for exclusion. Consider use of a flow diagram	3	Figure 1 and Figure 2
	b)	Report summary statistics for phenotypic exposure(s), outcome(s), and other relevant variables (e.g. means, SDs, proportions)	5	(Supplementary Table S1)
	c)	If the data sources include meta-analyses of previous studies, provide the assessments of heterogeneity across these studies	-	-
	d)	For two-sample MR:<br>i. Provide justification of the similarity of the genetic variant-exposure associations between the exposure and outcome samples<br>ii. Provide information on the number of individuals who overlap between the exposure and outcome studies	5	(Supplementary Table S1)
11	Main results			
	a)	Report the associations between genetic variant and exposure, and between genetic variant and outcome, preferably on an interpretable scale	5	Supplementary Table S1
	b)	Report MR estimates of the relationship between exposure and outcome, and the measures of uncertainty from the MR analysis, on an interpretable scale, such as odds ratio or relative risk per SD difference	5,7	Figure 2 and Figure 4
	c)	If relevant, consider translating estimates of relative risk into absolute risk for a meaningful time period	5,7	Figure 2 and Figure 4

	d)	Consider plots to visualize results (e.g. forest plot, scatter plot of associations between genetic variants and outcome versus between genetic variants and exposure)	6,8	Supplementary Figures S1-S5
12	Assessment of assumptions			
	a)	Report the assessment of the validity of the assumptions	4	No heterogeneity was detected in associations from three cytokine types, as evidenced by Cochrane's Q tests (both p-value of Q test from MR-Egger method and p-value of Q test from IVW method >0.05), while MR-PRESSO method identified no outlier SNPs. Additionally, the MR-Egger intercept disclosed no evidence of directional pleiotropy (p-value >0.05)
	b)	Report any additional statistics (e.g., assessments of heterogeneity across genetic variants, such as I^2, Q statistic or E-value)	7	Supplementary Table S2, S5, S8 and S11.
13	Sensitivity analyses and additional analyses			
	a)	Report any sensitivity analyses to assess the robustness of the main results to violations of the assumptions	7	Table 1
	b)	Report results from other sensitivity analyses or additional analyses	7	MR-PRESSO Global Test p-value
	c)	Report any assessment of direction of causal relationship (e.g., bidirectional MR)	7	Exploration of the causal effect of liver cirrhosis onset on inflammatory cytokines section
	d)	When relevant, report and compare with estimates from non-MR analyses	-	-
	e)	Consider additional plots to visualize results (e.g., leave-one-out analyses)	7	forest plots and leave-one-out sensitivity analyses of MR for the 41 cytokines in liver cirrhosis are in Supplementary Figure S1-S5.
	DISCUSSION			
14	Key results	Summarize key results with reference to study objectives	7	The inflammatory cytokine MCP-3 was suggestively associated with the greater risk of cirrhosis (odds ratio=1.332, 95% confidence interval: 1.012-1.752, p=0.041), while IP10 and IL-1RA were linked to a decreased risk. Furthermore, cirrhosis was significantly correlated with increased levels of several cytokines.

15	Limitations	Discuss limitations of the study, taking into account the validity of the IV assumptions, other sources of potential bias, and imprecision. Discuss both direction and magnitude of any potential bias and any efforts to address them	12	The lack of statistically significant associations for certain cytokines could be attributed to the limited sample size, which might have impacted the statistical power, or the potential influence of unaccounted-for confounding effects. Additionally, our study population primarily consisted of individuals of European descent, which may introduce ancestral bias. As a result, these conclusions may not be generalizable to non-European populations, and caution is needed when extending these findings to other ethnic groups. Furthermore, our investigation utilized data from two large-scale GWASs, and the absence of specific demographic information and clinical records for the study subjects precluded subgroup analyses. And the results of the MR study only represent changes in risk factors across the lifespan, rather than effects at a specific time after intervention. Therefore, caution should be exercised when applying MR results to clinical interventions. Further research is needed to validate our findings and assess their relevance in clinical diagnostic processes and therapeutic decision-making.
16	Interpretation			
	a)	Meaning: Give a cautious overall interpretation of results in the context of their limitations and in comparison with other studies		
	b)	Mechanism: Discuss underlying biological mechanisms that could drive a potential causal relationship between the investigated exposure and the outcome, and whether the gene-environment equivalence assumption is reasonable. Use causal language carefully, clarifying that IV estimates may provide causal effects only under certain assumptions	7	Observational studies have highlighted the pivotal role of inflammatory cytokines in cirrhosis progression. However, the existence of a causal link between inflammatory cytokine and cirrhosis remains uncertain. In this study, we conducted a bidirectional Mendelian randomization (MR) analysis at a summarized level to illuminate the potential causal relationship between the two variables. And we suggests that MCP-3 might be associated with the etiology of cirrhosis, while several inflammatory cytokines could potentially play a role in its downstream development. Additionally, the progression of cirrhosis was associated with elevated levels of HGF, suggesting a possible role for liver repair functions. However, as this is a preliminary study, further research is needed to confirm these findings and clarify the underlying mechanisms.

	c)	Clinical relevance: Discuss whether the results have clinical or public policy relevance, and to what extent they inform effect sizes of possible interventions		
17	Generalizability	Discuss the generalizability of the study results (a) to other populations, (b) across other exposure periods/timings, and (c) across other levels of exposure	7	Thirdly, as the study relied on a European database, the findings may not be generalizable to other ethnic groups, thus limiting the applicability of our results. Finally, a looser threshold was used to evaluate results, which might have increased false positives but also allowed for a more comprehensive assessment of the strong associations between cytokines profiles and liver cirrhosis.
	OTHER INFORMATION			
18	Funding	Describe sources of funding and the role of funders in the present study and, if applicable, sources of funding for the databases and original study or studies on which the present study is based	8	This research was funded by the National Natural Science Foundation of China, grant numbers 82372319, 81873979 and 81401666, the Ministry of Science and Technology of the People´s Republic of China, grant number 2021YFC2009306 and the Natural Science Foundation of Sichuan Province, grant number 2022NSFSC0736.
19	Data and data sharing	Provide the data used to perform all analyses or report where and how the data can be accessed, and reference these sources in the article. Provide the statistical code needed to reproduce the results in the article, or report whether the code is publicly accessible and if so, where	8	The datasets presented in this study can be found in online repositories. The names of the repository/repositories and accession number(s) can be found in the article/Supplementary Material.
20	Conflicts of Interest	All authors should declare all potential conflicts of interest	8	The authors declare that the research was conducted in the absence of any commercial or financial relationships that could be construed as a potential conflict of interest.

This checklist is copyrighted by the Equator Network under the Creative Commons Attribution 3.0 Unported (CC BY 3.0) license.

1. Skrivankova VW, Richmond RC, Woolf BAR, Yarmolinsky J, Davies NM, Swanson SA, et al. Strengthening the Reporting of Observational Studies in Epidemiology using Mendelian Randomization (STROBE-MR) Statement. JAMA. 2021;under review.

2. Skrivankova VW, Richmond RC, Woolf BAR, Davies NM, Swanson SA, VanderWeele TJ, et al. Strengthening the Reporting of Observational Studies in Epidemiology using Mendelian Randomisation (STROBE-MR): Explanation and Elaboration. BMJ. 2021;375:n2233.
